# The ASD Living Biology: from cell proliferation to clinical phenotype

**DOI:** 10.1038/s41380-018-0056-y

**Published:** 2018-06-22

**Authors:** Eric Courchesne, Tiziano Pramparo, Vahid H. Gazestani, Michael V. Lombardo, Karen Pierce, Nathan E. Lewis

**Affiliations:** 1 Autism Center of Excellence, Department of Neuroscience, University of California, San Diego, 8110 La Jolla Shores Drive, Suite 201, La Jolla, CA 92037 USA; 2 Department of Pediatrics, University of California, San Diego, 9500 Gilman Drive, La Jolla, CA 92093 USA; 3 Department of Psychology, Center for Applied Neuroscience, University of Cyprus, Nicosia, Cyprus; 4 Autism Research Centre, Department of Psychiatry, University of Cambridge, Cambridge, UK; 5 Department of Bioengineering, University of California, San Diego, 9500 Gilman Drive, La Jolla, CA 92093 USA; 6 Novo Nordisk Foundation Center for Biosustainability at University of California, San Diego, 9500 Gilman Drive, La Jolla, CA 92093 USA

**Keywords:** Autism spectrum disorders, Prognostic markers, Neuroscience

## Abstract

Autism spectrum disorder (ASD) has captured the attention of scientists, clinicians and the lay public because of its uncertain origins and striking and unexplained clinical heterogeneity. Here we review genetic, genomic, cellular, postmortem, animal model, and cell model evidence that shows ASD begins in the womb. This evidence leads to a new theory that ASD is a multistage, progressive disorder of brain development, spanning nearly all of prenatal life. ASD can begin as early as the 1st and 2nd trimester with disruption of cell proliferation and differentiation. It continues with disruption of neural migration, laminar disorganization, altered neuron maturation and neurite outgrowth, disruption of synaptogenesis and reduced neural network functioning. Among the most commonly reported high-confidence ASD (*hcASD*) genes, 94% express during prenatal life and affect these fetal processes in neocortex, amygdala, hippocampus, striatum and cerebellum. A majority of *hcASD* genes are pleiotropic, and affect proliferation/differentiation and/or synapse development. Proliferation and subsequent fetal stages can also be disrupted by maternal immune activation in the 1st trimester. Commonly implicated pathways, PI3K/AKT and RAS/ERK, are also pleiotropic and affect multiple fetal processes from proliferation through synapse and neural functional development. In different ASD individuals, variation in how and when these pleiotropic pathways are dysregulated, will lead to different, even opposing effects, producing prenatal as well as later neural and clinical heterogeneity. Thus, the pathogenesis of ASD is not set at one point in time and does not reside in one process, but rather is a cascade of prenatal pathogenic processes in the vast majority of ASD toddlers. Despite this new knowledge and theory that ASD biology begins in the womb, current research methods have not provided individualized information: What are the fetal processes and early-age molecular and cellular differences that underlie ASD in each individual child? Without such individualized knowledge, rapid advances in biological-based diagnostic, prognostic, and precision medicine treatments cannot occur. Missing, therefore, is what we call ASD Living Biology. This is a conceptual and paradigm shift towards a focus on the abnormal prenatal processes underlying ASD within each living individual. The concept emphasizes the specific need for foundational knowledge of a living child’s development from abnormal prenatal beginnings to early clinical stages. The ASD Living Biology paradigm seeks this knowledge by linking genetic and in vitro prenatal molecular, cellular and neural measurements with in vivo post-natal molecular, neural and clinical presentation and progression in each ASD child. We review the first such study, which confirms the multistage fetal nature of ASD and provides the first in vitro fetal-stage explanation for in vivo early brain overgrowth. Within-child ASD Living Biology is a novel research concept we coin here that advocates the integration of in vitro prenatal and in vivo early post-natal information to generate individualized and group-level explanations, clinically useful prognoses, and precision medicine approaches that are truly beneficial for the individual infant and toddler with ASD.

## Introduction

Autism spectrum disorder (ASD) has captured the attention of scientists and clinicians, as well as the lay public, in part because the clinical profile is so striking [1–3]. Affected individuals may display reduced ability to perceive, and engage in, every day social behaviors; they may display a preoccupation with seemingly trivial aspects of the environment (e.g., a fascination with street signs or spinning objects) and show challenges in expressing themselves verbally. Furthermore, there is considerable heterogeneity in each of those domains. Some individuals with ASD attempt to make friends and have good eye contact, while others appear completely aloof; some have IQs that far exceed normal average, while others are unable to talk [4]. Despite this interest and knowledge about ASD clinical heterogeneity, the prenatal beginning stages of ASD in the vast percentage of affected children are unstudied and unknown. This is because most current approaches are unable to examine the underlying abnormal fetal processes in the child with ASD that lead to that child’s clinical and neural outcomes. Thus, the prenatal and early post-natal biological bases for the striking clinical heterogeneity remain largely a mystery and controversial. Without this understanding, “precision” medicine and treatments at the individual child level do not exist in any meaningful way.

These major gaps are due to too little knowledge of what we term ASD Living Biology: We use the term ASD Living Biology to emphasize the critical need for the field to acquire an integrated system of knowledge about an ASD child’s development from abnormal prenatal beginnings to early clinical stages, arguably the most important developmental period in ASD. To gain that knowledge requires a paradigm shift that acquires and integrates genetic and in vitro prenatal molecular, cellular and neural measurements with in vivo post-natal neural and clinical presentation and progression in each ASD child. Thus, ASD Living Biology is a research concept we coin here that advocates the integration of within-child in vitro prenatal and in vivo post-natal information to generate individualized and group level explanations, clinically useful prognoses, and effective treatments for ASD. This ASD Living Biology approach enables individualized explanations and clinically useful prognosis and treatment. Such knowledge may lead to “precision medicine” for ASD: individualized early-age prognostic and treatment approaches. To specify such a system, we must address fundamental questions: When does autism begin? What are the initial molecular, neural, and developmental perturbations and their subtypes? How is neural development altered and what are the clinical implications of these neural developmental changes? Across the past decades, the answers to these questions were largely speculative and controversial.

**Fig. 1:**
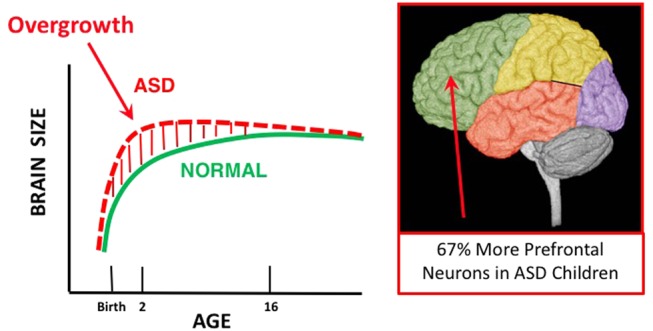
Illustration of early brain overgrowth in ASD. Brain overgrowth in the first years of life occurs in many ASD toddlers and is due to prenatal cell cycle dysregulation that causes an overabundance of cortical neurons. This is theorized to lead to disrupted neural network development and function, and ASD symptoms [5–7, 24, 31–34]. Unbiased, blinded stereological analyses find that young ASD male children have an average 67% more prefrontal neurons than controls [24]. Since cortical neuron generation occurs only in prenatal life in humans, this is direct evidence that ASD begins in the womb. As discussed in this review and previously [56], abnormal early brain undergrowth in toddlers with ASD may also be due to cell cycle dysregulation. Adapted from Courchesne et al. [7]

An attempt to answer some of these questions stemmed from our early brain overgrowth theory that posited that ASD results from a prenatal disruption of neurogenesis that generates an overabundance of cortical neurons and disrupts circuit formation and function in large-scale frontal, temporal and amygdala social and language networks [5–7] (Fig. 1). This theory resulted from the discovery of enlarged brain volumes in frontal and temporal cortex in many ASD toddlers and young children as evidenced from magnetic resonance imaging (MRI) [5, 8–14], increased cortical surface area or thickness during early-age development [15–18], increased volume of frontal axon tracts [19], accelerated and atypical head circumference growth across early life [20–22], and greater brain weight in ASD [23–25]. While it is also known that a subset of ASD have small brain size, sometimes associated with extremely rare chromosomal defects [26–29], a meta-analysis by Sacco et al. [30] of 8,310 subjects from 44 MRI and 27 head circumference studies spanning toddlerhood to adulthood finds significant brain and head circumference overgrowth in ASD compared to controls across ages; the most pronounced brain and head size increases occur at early ages.

**Fig. 2:**
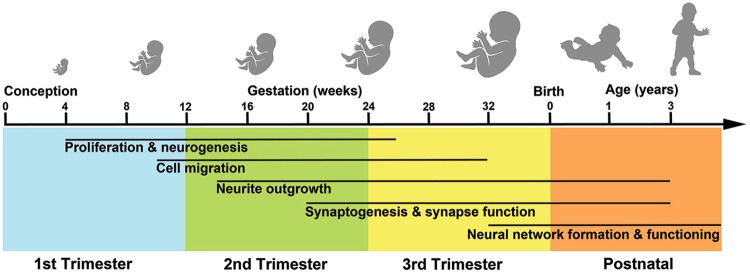
Developmental timeline relevant to ASD. Schematic of prominent processes occurring during different periods of human fetal and post-natal brain development. Adapted from Lombardo et al. [127]

Many now hypothesize that prenatal processes, such as abnormal cell proliferation, might underlie early-age brain growth defects in ASD [5–7, 24, 31–34], including both ASD overgrowth and undergrowth. Mechanisms regulating cerebral cell proliferation are well understood [35], and in humans, this fetal stage occurs in the 1st and 2nd trimesters (Fig. 2) [36–39]. Disruption of proliferation can have downstream consequences for differentiation, cell fate, migration, maturation, synapse development and circuit patterns. Therefore, it is crucial to know whether disrupted development in ASD begins as early as the proliferation stage, and to know if this represents a small subset of ASD or is more generally present.

Here we review recent postmortem, cell model, transcriptomic, genetic, genomic systems and animal model evidence that directly speaks to this specific theory of ASD beginnings and to the general hypothesis that ASD involves disruption of multiple fetal stages. We conclude that new evidence supports a new theory that ASD is a progressive prenatal disorder that precedes and produces post-natal phenotypic heterogeneity. As such, we also conclude that a paradigm shift toward an ASD Living Biology approach is necessary to uncover the beginning stages of ASD in each living child, understand how early neurobiological processes lead to the striking clinical heterogeneity in ASD, and develop “precision” treatments at the individual child level.

## ASD begins in the womb

### ASD postmortem evidence

Strong evidence that ASD begins in the womb comes from observations that young ASD male children have 67% more prefrontal neurons than controls [24] (Fig. 1). Neuron overabundance is not uniform in prefrontal cortex: Neuron excess was 79% in dorsolateral and 29% in medial prefrontal cortex [24]. Moreover, the degree of neuron excess across ASD cases was heterogeneous, ranging from 12 to 106%. In four ASD cases [24], neuron excess was >83% of control average. One child had an excess of 106% and mutation of *PTEN*, a high-confidence ASD gene. In another study, cortex in young ASD cases had 36% more neurons than controls [40] (Table S1). Young ASD males have ~53% more von Economo neurons in frontoinsular cortex than controls [41]. Cell cycle, differentiation, and DNA damage response genes and pathways are dysregulated in prefrontal cortex at young ages in ASD, and immune gene expression is upregulated [42]. Because proliferation of cortical neurons is exponential between 10 and 20 weeks of gestation [43, 44] and does not occur postnatally, pathological neuron excess cannot be caused by post-natal events and instead indicates neural disorder in ASD has a prenatal origin, likely by the first and/or second trimester.

In ASD, neural migration and cortical laminar organization, stages that occur in second and third trimesters, are also abnormal (Fig. 2) [34, 45–48]. Patches of focal laminar disorganization and nearby clusters of mis-migrated neurons were found in prefrontal and temporal cortices in 10 of 11 young ASD cases [34]. In these cases, there was heterogeneity in cortical cell types and layers that were most disrupted. While all layers were affected, patches tended to be pronounced in layers 2, 3 and 4. Patches of cortical dysplasia could be due to somatic mutations [49]. Somatic mutations (e.g., in the PI3K/AKT/mTOR pathway) also occur in hemi-megaencephaly and intractable epilepsy and may be an important prenatal source of phenotypic heterogeneity and risk in ASD and other neurodevelopment disorders [50].

Neuropathology examination of 4-year-old to 60-year-old ASD brains shows evidence of multiregional dysregulation of neurogenesis and neuronal migration in several cases, including subcortical, periventricular, hippocampal and cerebellar heterotopias reflecting abnormal neuronal migration and multifocal cerebral dysplasias [45]. Complete removal of the subplate, which normally occurs in the third trimester, may also be disrupted in ASD [47, 51]. Reduced growth of neuronal cell size and dendritic arbors in ASD postmortem cases have long been reported [25, 52] and are now recognized as an important neuropathological attribute of ASD cortex [46]; neuron size reduction varies from −4% in occipital to −18% in prefrontal cortex [24, 40, 53].

### ASD patient-derived iPS cells

**Fig. 3:**
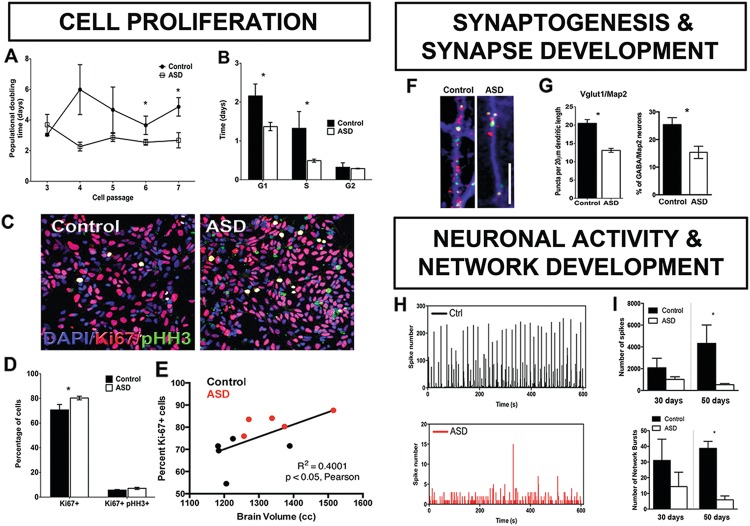
Excess cell proliferation, abnormal synaptic development and reduced neural activity are associated with iPS cells of ASD toddlers with enlarged brains. **a**  ASD iPS cells proliferate more rapidly than control. The iPS cells from ASD and control were differentiated to NPCs. From passages 2 to 6, cells were plated at the same density and population doubling time at each passage was calculated. Results of all lines (2 clones per line) are presented as mean ± s.e.m. (*repeated measurements *P* = 0.02, post hoc *P* < 0.04). **b** ASD cell cycle has abnormally short G1 phase. Adherent monolayer NPCs from control and ASD iPS cells were dissociated, counted for calculation of population doubling time and prepared for cell cycle analysis. Results are presented as the time spent in each cell cycle stage (*n* ⩾ 4, mean ± s.e.m., analysis of variance (ANOVA) *P* < 0.04, post hoc *P* < 0.04 for comparing the time spent in G1 phase in the ASD NPCs with those of the control NPCs, respectively). **c** Control and ASD NPCs were immunostained with 4′,6-diamidino-2- phenylindole (DAPI; blue), anti-pHH3 (green) and anti-ki67 (red) (scale bar: 200 μm). Representative images of the staining are shown. **d** Quantification of the percentage of Ki67^+^ − and Ki67^+^ pHH3^+^ −labeled cells are presented as mean ± s.e.m. (*n* ⩾ 5; *P < 0.03 for comparing the results of the ASD with those of the control NPCs). **e** Greater proliferation rates in ASD were correlated with greater early brain overgrowth. Pairwise correlation between individual brain size (volume) and respective NPC cell line proliferation rates (% of Ki67-positive cells). ASD displays reduced and deviant synaptic development as shown in panels f and g.  **f** Representative images of synaptic processes from cells after neuronal differentiation (Map2, blue). The iPSC-derived neurons express markers for excitatory neurons, such as postsynaptic density protein 95 (PSD95, red) and vesicular glutamate transporter 1 (VGlut1, green) (scale bar: 5 μm). **g** Bar graphs show synaptic puncta size in ASD vs control neurons (*P* < 0.05 for comparing the results of the ASD with those of the control neurons), and GABA-positive neurons in all ASD-derived neurons compared with all controls (*P < 0.001). **h** ASD neural activity is sharply reduced. Representative image of number of spikes recorded over 10 min at 50 days of culture maturation (*n* = 3 wells per cell type). **i**  ASD neural activity is sharply reduced. Top, total number of spikes from data obtained from controls (*n* = 6) and ASD (*n* = 10) clonal lines differentiating over 30 days and controls (*n* = 4) and ASD (*n* = 9) clonal lines at 50 days after differentiation over 10 min of recording. Results are presented as mean ± s.e.m. (*P = 0.0046 for comparing the results of the ASD with control networks). Bottom, number of network bursts from wells that were able to generate bursts (10 spikes over 100 ms). Results are presented as mean ± s.e.m. (*P < 0.0001 for comparing the results of the ASD with control networks). Images adapted from Marchetto et al. [54]

To test whether disruptions in proliferation and prenatal neural developmental stages are related to early brain overgrowth in vitro in ASD, Marchetto et al [54] derived iPS cells from fibroblasts from living ASD toddlers with early brain overgrowth and from control toddlers. iPS cells were differentiated to neural progenitor cells and then neurons. ASD neural progenitor cells display excess proliferation compared to normal controls (Fig. 3a–d), and that excess proliferation was correlated with the degree of each child’s MRI-based brain overgrowth (Fig. 3e). This suggests dysregulated proliferation contributes to early brain overgrowth in ASD toddlers. Excess proliferation of iPS cells and radial glia cell stage and cell cycle acceleration were also found by Mariani et al [55] in iPSCs derived from a small sample of ASD patients with brain enlargement. In Marchetto et al. [54], ASD neural progenitors doubled in number twice as fast compared to controls due to a shorter cell cycle G1/S phase. Further, reduced β-catenin and BRN2 transcriptional activity was related to this proliferation abnormality. There was also earlier but less complete cell differentiation.

Multiple subsequent fetal age processes were also abnormal in every ASD case in Marchetto et al [54]. ASD patient-derived neurons additionally displayed fewer excitatory synapses, and downregulation of multiple neurotransmitter and synapse markers including GABA receptor markers (Fig. 3f, g). With increasing age from 30 to 50 days of development, ASD-derived neural networks showed less spontaneous excitatory activity and poorly synchronized activity. By 50 days, synchronized neural bursts of activity were 6 times lower in ASD than controls (Fig. 3h, i). The ASD subject with the greatest cell proliferation and brain size has a *PTEN* mutation and is not intellectually disabled but has severe social communication symptoms. Mariani et al. also reported multiple disrupted fetal stages in their ASD iPSC study [55], and interestingly these included excessive synaptogenesis. Thus, ASD patient-derived iPSC studies find evidence of multiple fetal stage defects with commonality in excess proliferation and cell cycle acceleration defects, but heterogeneity in synaptogenesis effects. This points to fetal stage subtypes in ASD.

These ASD Living Biology experiments suggest that neural developmental disorder in ASD spans prenatal and early post-natal life, beginning with dysregulation of cell cycle G1/S and excess cell proliferation and ending with disrupted synaptogenesis and dysfunctional nascent neural circuit activity. Heterogeneity already exists at these early stages. Mechanisms that dysregulate proliferation may dysregulate neural differentiation, cell fate determination and maturation. The conclusion that dysregulation of prenatal cell cycle G1/S underlies excess proliferation and early brain overgrowth in these toddlers with ASD is strongly supported by these ASD patient-derived iPS cell data.

### Transcriptomic evidence in living ASD toddlers points to prenatal periods

Transcriptomics of leukocytes from ASD toddlers reveal further insights into ASD Living Biology. In analyses of gene expression and MRI measures of brain size at young ages, ASD toddlers with the most abnormal expression of cell cycle hub genes had the most brain overgrowth, while those with milder cell cycle disruption had smaller than normal brain size [56]. Functional genomic analyses identified 23 candidate genes for brain maldevelopment linked to 32 upstream high-confidence ASD genes [56]. Dysregulation of cell cycle G1/S was identified as contributing, suggesting shortened G1/S in bigger ASD brains, consistent with Marchetto et al [54]. *CHD8* is one of the most commonly found high-confidence ASD mutations [57, 58, 62, 66], and the analyses of a CHD8 subnetwork and altered CHD8-regulated transcript levels further confirmed the central role of genes regulating neurogenesis and cell adhesion processes in ASD brain maldevelopment [56]. Thus, in living ASD infants and toddlers, patient-derived iPS cell experiments and leukocyte transcriptomics point to dysregulation of cell cycle G1/S.

### High-confidence ASD genes and prenatal development

In a small percentage of children with ASD, rare de novo gene mutations are risk factors [58–65]. Genetic information accumulated via these and many other studies enable the search for likely ASD genes and gene networks. Unraveling how these mutations affect brain development is crucial to advance understanding of how genetic, non-genetic and gene/environment interactions cause ASD and influence neural development, clinical progression, and treatment. However, new evidence shows many proposed gene associations may be “noise” [66]. Of the hundreds of potential associations, 38 genes are classified as recurrent and potentially penetrant in ASD [66]. Of these 38 genes, 31 also overlap with the 65 SFARI Level 1(S) and 2(S) *ASD* genes; taken together, these 72 genes are high-confidence ASD (*hcASD*) genes.

These 72 *hcASD* genes, many of which regulate gene expression, are the most penetrant genes implicated in ASD thus far. It is crucial to understand how they contribute to brain growth and function because they may point to processes central to ASD development. Previous analyses of ASD risk genes were performed when knowledge of *hcASD* genes was more limited [67, 68], and some of those analyses gave similar weight to both low and high-confidence genes. Thus, the developmental roles of these 72 *hcASD* genes may have been obscured by inclusion of low confidence genes; so further study of these *hcASD* genes is needed.

Here we use these 72 *hcASD* genes to address three central questions: When during development do *hcASD* genes play developmental roles? Where in the brain do they do so? What functional roles may they play? First, we addressed the “when and where” roles of these *hcASD* genes by leveraging the most recent BrainSpan RNA-Seq dataset (http://www.brainspan.org/). Of the 72 genes, 3 are not detected (RPKM > 0.5) in >10% of BrainSpan samples between prenatal and up to 8 years old post-natal. Therefore, here we focus on the 69 *hcASD* genes that do have reliable expression levels.

**Fig. 4:**
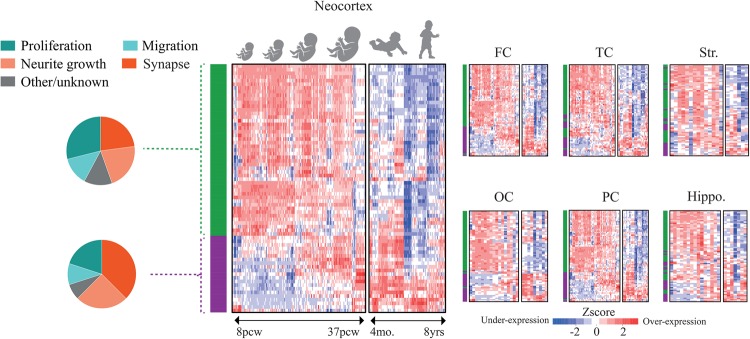
The majority of *hcASD* genes show peak expression during prenatal life in different brain regions. Heatmaps demonstrate the developmental expression patterns (*x-*axis) of 69 *hcASD* genes (*y*-axis) in different brain regions in prenatal and post-natal development. In the large neocortex heatmap, hierarchical clustering of neocortex developmental transcriptome reveals two main clusters of genes, one cluster displayed in green and the other in purple (see y-axis of heatmap). Proliferation and neurogenesis *hcASD* genes make the largest contribution to the green cluster, and synapse development and function *hcASD* genes make the largest contribution to the purple cluster; see the two pie charts on the far left. This clustering pattern is present across different neocortex regions and, to a lesser degree, in hippocampus and striatum (see green and purple *hcASD* gene cluster patterns on y-axis of the other six heatmaps). FC: frontal cortex, Hippo: Hippocampus, OC: occipital cortex, PC: parietal cortex, Str: striatum, TC: temporal cortex.

Of these 69 genes, 65 (94%) are expressed (RPKM > 1) in prenatal brain development. Importantly, 47 genes (68%; green cluster in Fig. 4) have peak expression during much of prenatal development, and then are downregulated following birth. Furthermore, they display peak expression in brain regions that are abnormal in ASD, including major cerebral cortical areas, hippocampus, striatum (Fig. 4), and cerebellum. From the perspective of biological roles, these *hcASD* genes are largely associated with proliferation and differentiation. Many are also involved in neural migration, neurite outgrowth, and early synaptogenesis (Fig. 4). The remaining 22 (32%) of the *hcASD* genes start to increase in expression levels during the 3^rd^ trimester and the first several years of post-natal life (Fig. 4; purple cluster). They also express in multiple, widespread brain systems known to be abnormal in ASD (Fig. 4).

**Fig. 5:**
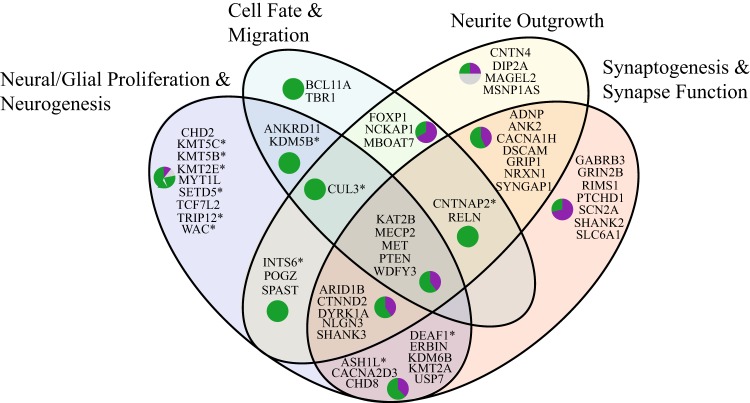
Distribution of 58 *hcASD* genes in four main categories of neural development. Functional annotation could be found for 58 *hcASD* genes based on a manual literature search. Most highly penetrant ASD genes are pleiotropic, being involved in multiple stages of brain development. The small pie-charts in each region indicate the percentage of genes in green and purple clusters from Fig. 4. The gray color in pie-charts represents percentage of genes with no strong expression level in fetal and early post-natal periods. A gene is marked by an asterisk  (*) if its function was inferred from evidence in adult neural stem cells, embryonic or hematopoietic stem cells, central nervous system other than brain, or, for one gene, cancer (see Table S2 for details and references).

Second, we addressed “what” developmental role all 72 *hcASD* genes play, and found studies that support involvement of 58 out of 72 *hcASD* genes in at least one of the four main brain development processes including proliferation/neurogenesis, cell fate/migration, neurite outgrowth, and spine development/synaptogenesis/synapse function (Table S2). Of these 58 *hcASD* genes, 33 (57%) *hcASD* genes are involved in cell proliferation and/or neurogenesis, while 34 (59%) are involved in spine development, synaptogenesis, and/or synapse function (Fig. 5). Importantly, our analysis underscores a crucial feature shared by the majority of *hcASD* genes, which is functional pleiotropy. Indeed, 36 out of 58 (62%) *hcASD* genes are involved in multiple major developmental processes (Fig. 5). While *hcASD* genes have been historically described to play primary roles in synaptic development and function, it is becoming increasingly clear that this view may represent only a partial understanding of how they contribute to ASD pathophysiology, which begins earlier during development. For example, *MECP2* is known as critical player for synapse development and function of GABA-releasing neurons [69, 70]; however, its involvement spans early stages of cortical cell proliferation (likely enhanced proliferation in ASD) [71, 72, 73], neurogenesis and neural migration [73, 74], and neurite outgrowth [75]. *SHANK3*, a prototypical *ASD* gene coding for a postsynaptic scaffolding protein in excitatory synapses [76], also contributes to neurogenesis and morphogenesis during early brain development [77].

While our analysis highlights the roles of several *hcASD* genes, it is expected that further work will continue to propose and validate new genes. For example, a recent machine learning approach leveraged previously implicated genes to generate predictions of plausible new autism risk genes [78]. They further demonstrated that these genes converge on fetal and early infancy stages, and were involved in processes including cell cycle, embryogenesis,  morphogenesis, axonogenesis and synaptogenesis [78]. Furthermore, these genes also affect multiple brain structures, and are implicated in G1/S, RAS/ERK-PI3K/AKT signaling, and chromatin remodeling.

In summary, our analyses of *hcASD* genes (Fig. 4) and emerging work on newly predicted ASD risk genes highlight two major human neural development epochs. First, these genes are highly active during early fetal stages of cell proliferation, differentiation, migration and early organization. Second, they impact late fetal generation of synapses and functional assemblies that continue on through birth and impact the early experience and learning years. Thus, *hcASD* gene evidence dovetails neatly with ASD patient-derived iPS cell and leukocyte transcriptomic evidence in ASD toddlers, and ASD postmortem evidence reviewed above.

## Pleiotropic common pathways implicated in ASD

**Fig. 6:**
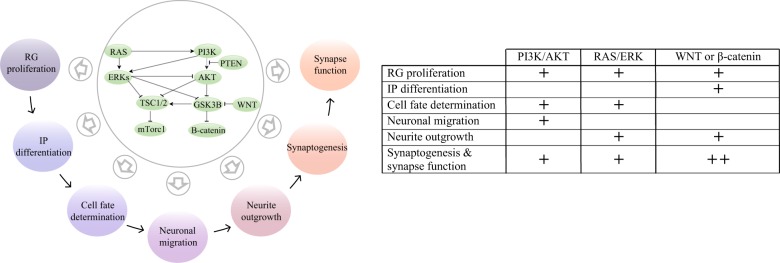
The RAS/ERK, PI3K/AKT, WNT and β-catenin signaling pathways are involved in different stages of brain development and are commonly disrupted in ASD. The schematic on the left indicates that these signaling pathways are highly interconnected and modulate different aspects of brain development. For this illustration, the pathways are simplified and some intermediate genes are not shown. In the table on the right, a plus sign  (+) for each pathway indicates that its dysregulation has been reported in the corresponding fetal developmental stages based on studies on either *hcASD* genes, ASD-derived neurons, or both (double plus signs).

Among the *hcASD* genes, at least 48 (67%) genes are involved in gene regulation including chromatin structure, transcription, translation, and post-translation stages. This suggests that perturbations of gene regulation contribute to ASD. Consistently, studies on *hcASD* genes and iPSC-derived ASD neurons highlight signaling pathways that are commonly disrupted, such as PI3K/AKT [79, 80], RAS/ERK [79, 80, 82, 83], WNT and β-catenin [57, 83, 84]. These signaling pathways are highly interconnected with crosstalk and convergence points (Fig. 6) that can vary based on the cell context and strength of input signal [85–87]. In neural development, these pathways are crucial in different fetal and early post-natal stages (Fig. 6; Table 1), suggesting that the pleiotropic concept can be extended from genes to molecular pathways.

**Table 1:** Examples of *hcASD* genes that converge on and disrupt RAS/ERK, PI3K/AKT, WNT and β-catenin signaling pathways

Gene name	Pathway involved	Examples of effects on pathways
*FMR1* ^b,c,d^	PI3K/AKT	*Fmr1* knock-out mice have elevated activation of PI3K/AKT signaling pathway, affecting synaptic plasticity with behavioral and cognitive defects. Downregulation of PI3K in prefrontal cortex of *Fmr1* null mice ameliorates aberrations in protein synthesis, dendritic spine density and cortical network activity.
*FOXG1* ^b^	PI3K/AKT	The PI3K/AKT signaling pathway affects cell fate determination and neural migration by regulating *FOXG1*.
*NLGN3* ^a,c,d^	PI3K/AKT	*NLGN3* secretion by active neurons enhances proliferation of glial cells by activation of PI3K/AKT pathway. Also affects synaptic development.
*PTEN* ^a,b,c,d^	PI3K/AKT	*Pten*^+/-^ mice show high activity in PI3K/AKT and β-catenin signaling resulting in enhanced proliferation of radial glial cells and cortical overgrowth; neuronal hypertrophy, hypertrophic and ectopic dendrites and axonal tracts; increased synapses; and hyperconnectivity of prefrontal cortex with amygdala. *Pten* also involves in neural migration.
*RELN* ^b,c,d^	PI3K/AKT	PI3K/AKT signaling pathway affects cell fate determination and neural migration by regulating *RELN*. While *RELN* promotes neuronal maturation, synaptic formation and plasticity, it appears to be more important for dendrite and spine development via PI3K/AKT/mTOR pathway.
*SHANK3* ^a,c,d^	PI3K/AKT	Mice with *Shank3* mutations show deficits in synaptic function, hippocampal LTP and motor performance that can be improved with IGF1 treatment. Also affects neurogenesis.
*MECP2* ^a,b,c,d^	PI3K/AKT and RAS/ERK	iPS-derived RG cells from Rett syndrome patients with *MECP2* mutation exhibit excess proliferation through activation of PI3K/AKT and downregulation of RAS/ERK pathways. Neurons with *MECP2* knock-down fail to attain a unipolar/bipolar shape, display abnormal migration and reduced cortical thickness. Neuron models of *MECP2* mutations lead to abnormalities in soma size, dendritic arborizations, spine density, and neuronal firing that may be partially rescued by inhibiting *Pten* gene or IGF1 treatment.
*ARID1B* ^a,c,d^	PI3K/AKT, WNTand β-catenin	*ARID1B* suppression delays cell cycle re-entry. Mice with *Arid1b* knock-down show suppressed IGF1, a mediator of PI3K/AKT and β-catenin pathways, resulting in decreased dendritic arborization and accumulation of aberrant dendritic spines and altered synaptic transmission.
16p11.2^a,d^	RAS/ERK	16p11.2 is one of most recurrent CNVs in ASD and encompasses multiple genes including *ERK1*. Its deletion dysregulates RAS/ERK with effect on proliferation and neurogenesis of murine neural progenitor cells. 16p11.2 mutation mouse models show reduced RAS/ERK activity, reduced protein synthesis and cognitive impairments.
*ERBIN* ^a,d^	RAS/ERK	*ERBIN* has an inhibitory effect on RAS/ERK signaling pathway. Upregulation or downregulation of *Erbin* leads to enhanced or decreased differentiation of PC12 neurons, respectively.
*KAT2B* ^a,b,c,d^	RAS/ERK	Null mutations lead to dysregulation of RAS/ERK and disruption of the pyramidal cell layer organization.
*NF1* ^d^	RAS/ERK	Mice with *Nf1* null mutations show dysregulated RAS/ERK pathway and deficits in spine morphology, glutamate and GABA release, hippocampal LTP and learning abilities.
*SYNGAP1* ^c,d^	RAS/ERK	*SYNGAP1* acts as RAS inhibitor, and heterozygote mutations in mice show premature neurons, elevated excitatory synaptic transmission, reduced axonal branching and synaptic boutons in inhibitory neurons, and deficits in behavior and cognition. Rescuing *Syngap1* in adulthood did not benefit the mice, underscoring its role in prenatal and early post-natal brain development.
*CHD8* ^a,d^	WNT and β-catenin	*CHD8* knock-down disrupts G1/S phase, dysregulates proliferation in neural progenitor and stem cells and causes brain overgrowth. *Chd8* mutations in mice result in abnormalities in striatal circuitry and synaptic physiology
*CTNND2* ^a,c,d^	WNT and β-catenin	Affects proliferation of glioma cells and involved in glioblastoma. Regulates spine morphology; mutations decrease spines and excitatory synapses in hippocampal neurons in rodents. *Ctnnd2* mutation in mouse models have impaired spatial learning and fear conditioning.

See Supplementary Table S3 for references to individual studies

Functional Roles in Development (also see Fig. 5)

^a^ Neural/Glial Proliferation and Neurogenesis

^b^ Neuronal Fate Determination and Migration

^c^ Neurite Outgrowth

^d^ Synaptogenesis and Synapse Function

### Dysregulation of proliferation and neurogenesis

Multiple ASD risk factors, including *PTEN*, *MECP2*, *CHD8*, *ARID1B*, *ERBIN*, and the 16p11.2 locus, link ASD to the disruption of neuron production through the PI3K/AKT, RAS/ERK, WNT and β-catenin pathways (Table 1). In precursor and radial glial cells (RGCs), upregulation of PI3K/AKT, WNT and β-catenin or downregulation of RAS/ERK leads to elevated proliferation [86, 88, 89]. The role of these pathways reverses in intermediate progenitor cells (IPCs). Activity of β-catenin (downstream of PI3K/AKT and WNT, Fig. 6) enhances premature differentiation of neurons [88], while activity of RAS/ERK reduces neurogenesis of IPCs and increases astrogenesis [90]. During human brain development, precursors differentiate into neurons first and glial cells later [91]. Therefore, dysregulation of proliferating precursor cells will perturb the number of generated neurons as well as the balance between neurogenesis and gliogenesis in the developing brain. One of the primary mechanisms that regulate neuroprogenitor divisions is the rate of cell cycle progression, particularly G1/S transition time [92, 93]. Studies on *CHD8* and iPS-derived ASD neurons indicate the dysregulation of proliferation and macrocephaly in ASD could be linked to the disruption of these signaling pathways and shortened G1/S transition[54, 55, 57, 94, 183, 192, 193]. Importantly, the induced proliferation by the signaling pathways can result in either a thicker or thinner cortex in mice models depending on the strength of the dysregulation. While downregulation of RAS/ERK can lead to a thicker cortex from increased proliferation and an increased neuronal count [86], loss of *Erk2* can induce such strong G1/S dysregulation that mice cells miss the time window of neurogenesis (E14.5–E16.5), resulting in fewer neurons, precocious astrogenesis, and potentially microcephaly [95]. The other key factor that controls neuron numbers is the balance between cell cycle re-entry (proliferative divisions) and exit (neurogenesis). During brain development, RGCs can divide symmetrically or asymmetrically. While symmetric divisions result in two daughter RGCs, asymmetric divisions produce a RGC and one another cell [96]. The newly generated cell can differentiate into a neuron, or become an IPC [97]. IPCs can undergo 1–3 proliferation cycles before differentiation to neurons [97, 98]. Cortical surface area and thickness are controlled by the mechanisms involved in RGC and IPC proliferation and differentiation decisions [99, 100]. Interestingly, G1/S transition rates might be associated with these cell decisions as symmetric divisions can have shorter G1/S transition times than asymmetric divisions [93, 101]. Evidence suggests that PI3K/AKT, RAS/ERK, WNT and β-catenin pathways are major players of these cell decisions as well. RGCs in a mouse model of *Gsk3* knockout, a major convergence point of the signaling pathways (Fig. 6), show more highly active β-catenin and Notch pathways [102]. As a result, RGCs were locked in proliferative state with attenuated neurogenesis [102]. This resulted in expanded RGC pools, fewer IPCs, reduced cortical thickness and increased ventricular apical surface length [102]. Hence, it is tempting to speculate that perturbation of these four pathways in ASD could involve dysregulation of balance between proliferation and neurogenesis as well as neurogenesis and gliogenesis.

### Disruption of cell fate and migration

Studies on *MECP2*, *KAT2B*, *REELIN* and *FOXG1* *hcASD* genes suggest that they are associated with changes to the cell fate determination and neural migration processes in ASD through PI3K/AKT, RAS/ERK, WNT and β-catenin pathways (Table 1). Upregulation of WNT and β-catenin signaling, for example, prevents the multipolar to bipolar transition of pyramidal precursors [103]. Thus, neurons fail to migrate to the cortical plate and accumulate within the intermediate zone [88, 104]. Somatic mutations can also lead to dysregulation of these signaling pathways during brain development. Somatic activating mutations in the PI3K/AKT pathway are associated with hemimegalencephaly and focal cortical dysplasia syndromes that are marked by cortical dyslamination, dysmorphic neurons and loss of radial neuronal orientation [50, 105, 106]. Thus, in addition to modulating neuron development, the perturbed pathways in ASD also alter cell fate determination and neural migration.

### Dysregulation of neurite outgrowth and neuronal function

The *hcASD* genes *SHANK3*, *FMR1*, *CTNND2*, *CHD8*, *SYNGAP1*, *MECP2*, *PTEN*, *ARID1B* and *NF1* are associated with dysregulation of neurite outgrowth and neuronal function through the PI3K/AKT, RAS/ERK, WNT and β-catenin signaling pathways (Table 1). These pathways control soma size, dendritic arborization, axon generation, spine development and synapse function [184–188]. Activity of receptor tyrosine kinases, metabotropic glutamic receptors and NMDA receptors lead to the activation of PI3K/AKT and RAS/ERK signaling pathways [185, 189], and thereby influence synaptic plasticity by mediating expression and trafficking of AMPA receptors in excitatory synapses and controlling long-term depression (LTD) and long-term potentiation (LTP) responses [188–190]. PI3K/AKT and RAS/ERK signaling pathways also control processes associated with neural excitotoxicity and cell survival [191]. Interestingly, it seems that *hcASD* genes do not impact these pathways in the same way. For example, while mutations in *Fmr1* and *Pten* lead to the over-activity of PI3K/AKT pathway with increased synaptogenesis and hyperconnectivity of the neurons [107–109], mutations in *Mecp2* and *Shank3* are reported to suppress the pathway, leading to delayed and sparse spine development and reduced synaptic amplitude [110, 111]. Interestingly, in cases of *Mecp2* and *Shank3*, neurons could be partially rescued by activating PI3K/AKT pathway through *Pten* inhibition or IGF1 treatment, while downregulation of the pathway in *Fmr1* null mice ameliorated aberrations in protein synthesis, dendritic spine density and cortical network connectivity [108, 109].

Finally, by prenatal injection of a small molecule that stabilizes axin, a component of the WNT and β-catenin signaling pathway, cell cycle proliferation is prolonged and causes an excess of layer 2 and 3 neurons and early brain overgrowth, abnormal synaptogenesis and increased excitatory neural activity [112]. This was accompanied by ASD-like abnormal social, vocalization, and ritualistic behaviors. These results strengthen the causal link between cell cycle disruption and ASD and also suggest that non-genetic etiologies may underlie deviant neurodevelopmental trajectories.

Thus, RAS/ERK, PI3K/AKT, WNT and β-catenin are highly integrated and pleiotropic, exhibiting different and successive roles throughout prenatal and early post-natal life (Fig. 6). Across ASD individuals, variation in how and when these pleiotropic pathways are dysregulated will cause early heterogeneity of pathophysiology and potentially later neural and clinical heterogeneous outcomes.

## The prenatal maternal immune activation model of ASD

Non-genetic etiologies may account for 30–41% of the risk for ASD [113], and such factors may act independently or in combination with genetic factors [113]. A well-established non-genetic model system for studying ASD pathophysiology is the prenatal maternal immune activation (MIA) model [114–116]. The link between MIA and enhanced risk for ASD is bolstered by large-scale population-based studies showing small but significantly increased risk due to maternal prenatal infections [117–122]. MIA models have ASD-like social, vocalization, ritualistic, exploration and other behavioral deficits [123–125]. Current MIA ASD models expose developing rodent pups to bacterial or viral mimetics [126] to elicit strong immune responses. This is timed to roughly mimic infection between 8 to 12 weeks during the 1st trimester in humans, when neural progenitor cells are proliferating to expand cortical surface area and promote cortical layering [37, 39].

**Fig. 7:**
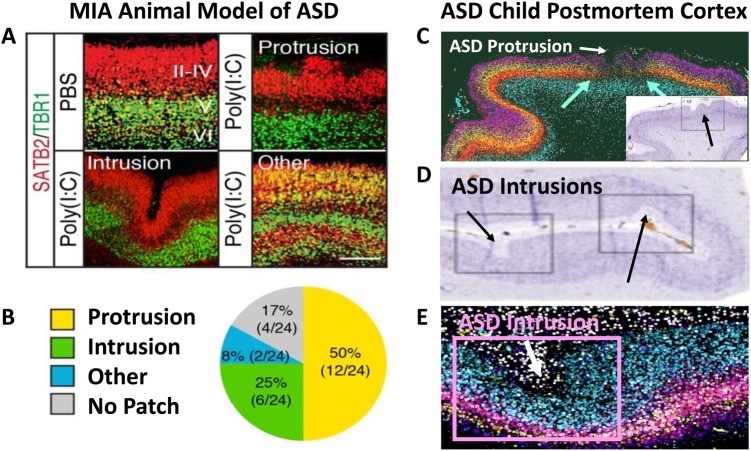
In a single litter, a single prenatal poly(I:C) injection may cause different cortical layering defects across pups that resemble some types of focal cortical dysplasias seen in different individuals with ASD.  **a** Expression of multiple layer-specific markers (II–IV and V, VI) in mouse cortex from Choi and colleagues [81, 132]. Typical mouse cortex layer development is shown in upper left corner (PBS), while heterogeneous types of focal cortical dysplasia are caused by prenatal maternal immune activation (MIA) by synthetic dsRNA, poly(I:C) as shown in the other three panels. **b** In a single litter, a single prenatal poly(I:C) injection may cause different layering defects across pups including protrusions, intrusions and laminar disorganization and other types of focal cortical dysplasia as well as pups with typical cortex. The cortical and ASD-like behavioral MIA-caused phenotypes are dependent on maternal IL-17a. **c** A 9 year old ASD postmortem case with *ADNP* gene mutation has focal macroscopic frontal cortical surface malformation (white arrow) and interruption of underlying cortical layering as visualized by expression of multiple layer-specific and cell type specific markers (see region marked by the two light blue arrows; see Fig. 1 in Stoner et al [34]). Different marker genes represented by different colors. This focal region also has clusters of mis-migrated cells (not shown in this section). Inset shows the same cortex location visualized by nissl staining to further reveal surface undulation and protrusion (see black arrow). A 2 year old ASD postmortem case shown in **d** with surface intrusions visualized by nissl staining and in **e** with surface intrusion visualized by multiple layer-specific and cell type specific markers [34]. Adapted from Choi et al. [132] and Stoner et al. [34]

MIA strongly upregulates cell cycle gene expression and cell proliferation, and may cause brain overgrowth [127–129]. Similar to some ASD cases, MIA induces overproduction of neurons [129], and increases cortical thickness [129, 130] and brain size [130] as seen in many toddlers with ASD [5, 8, 10, 13, 14, 18]. Gene expression alterations also involve neuronal migration processes [128] that could underlie observed cortical layering defects, characterized by a 24% increase in neuron numbers in cortical layers II/III [131]. MIA can also induce more complex cortical phenotypes such as focal cortical dysplasia [81, 132], similar to those found in ASD [34] (Fig. 7). Also, cytoarchitecture heterogeneity is seen that is characteristic of ASD cases [34, 132]. When focal cortical dysplasia is present, ASD-like behaviors occur; but when its development is prevented, ASD-like behaviors do not occur [81].

In addition to causing dysregulation of proliferation, abnormal neuronal migration and dysplasia, increased brain size and ASD-like behavior, MIA animal models also lead to many other neuroanatomical and molecular abnormalities found in ASD (Table 2).

**Table 2:** ASD-like molecular, cellular, anatomical and behavioral abnormalities in MIA models

Human development	Abnormalities induced by MIA and associated with ASD
1st–2nd Trimester	Upregulated cell cycle and downregulated migration and neurite outgrowth gene expression
	Cortical layering: over-production of neurons, increased cortical thickness, focal cortical dysplasia
	Cerebellar vermis dysplasia
2nd–3rd Trimester	Microglia: enhanced priming, activation
	Dendritic morphology abnormalities
	GABAergic signaling, excitatory/inhibitory imbalance, number of interneurons
3rd Trimester—post-natal	White matter neuron density
	Dendritic spines number and turnover rates
	Synaptic pruning and proteins
Post-natal	Early brain overgrowth
	Myelin functionality and stability
	Dopamine system
	Serotonin levels
	ASD-like abnormal social, vocalization, and ritualistic behaviors
	Gender-dependent effects
	Transgenerational effects

See Supplementary Table S4 for references to individual studies

Given the diverse ASD-associated phenotypes that arise with a prenatal immune challenge, MIA-induced effects on neurodevelopment provide insights into the molecular mechanisms behind ASD and related neurodevelopmental disorders. Within the ASD neuropathological and molecular heterogeneous landscape, MIA disrupts fetal development by involving transcriptional programs that are abnormal in adult ASD brain tissue and genes with high-confidence ASD mutations or their downstream targets [127]. This evidence suggests shared disrupted pathways between genetic and non-genetic etiologies and the possibility for a much more relevant interplay between a genetic background at-risk for ASD and environmental triggers. Work by Le Belle et al. [130] identified that brain enlargement due to *Pten* haploinsufficiency in mouse increased from 8 to 44% when combined with MIA, thus demonstrating the interactive effects of MIA and ASD gene mutations in the disruption of cortical development. Such augmented disruption occurs via the activation of the cellular redox signaling in response to infection [130,133]. Reactive oxygen species (ROS) can increase stem cell self-renewal and neurogenesis through the reversible inactivation of PTEN and hyper-activation of the NADPH oxidase (NOX)-PI3K pathway [134], which ultimately, during early post-natal development, will result in enlarged brains and autistic behaviors.

MIA also causes long-lasting deleterious effects on the post-natal brain similar to those expected from germline mutations in patients with neurodevelopmental disorders [127]. Such effects likely involve the epigenetic dysregulation of transcriptional programs that are sensitive to environmental changes or insults [135–137]. Inflammatory mediators (e.g., cytokines) induce such alterations during neurodevelopment [138] by histone modifications [139], methylation changes at the promoter of ASD-critical genes (e.g., *MECP2*) [140] or genes involved in synaptic functions [141] and global methylation changes [136]. Importantly, increased levels of IL-6 alone recapitulate aspects of ASD pathophysiology [138, 142, 143]. At the molecular level, IL-6 increases the activity of DNA methyltransferases 1 (DNMT1) with activating effects on the JAK/STAT3, NF-κB and PI3K/AKT/mTOR signaling pathways [144–148] affecting the regulation of autophagy-related functions, that are required to maintain cellular homeostasis under stress [149], such as a prenatal insult. This provides a strong link between pro-inflammatory immune factors, body growth through activation of the mTOR pathway and uncontrolled proliferation typical of cancer-related disease [150, 151–153]. Genetic or epigenetic dysregulation of such pathways is associated with several neurodevelopmental disorders, in particular psychiatric, characterized by abnormal brain size (enlarged and reduced) [154, 155]. Consistently, we found that MIA alters key components of the TSC/mTOR signaling pathway with ultimate activation of the translation initiation factor EIF4E which is predicted to alter the regulation of neural progenitor cell divisions during midgestation [127, 156–158]. Such regulation may also be altered by activation of microglia cells under stress conditions including MIA and/or in response to increase levels of IL-6 [142]. Impairment in the regulation of microglia functions is consistently seen in ASD studies [42, 159, 160] and is likely involved in abnormal regulation of neuron numbers both at pre- and post-natal stages.

Accumulating evidence consolidates the idea that environmental models of ASD-associated phenotypes may provide valuable insights to help develop potential treatment, prevention, or intervention strategies. For example, MIA alters expression in pathways shared with those in fragile X syndrome for which advances in drug development are in progress. With a better understanding of mechanisms behind environmental etiologies of ASD, such as MIA, we could potentially identify therapeutic targets amenable to prevention and/or treatment later in life [161, 162]. In this scenario, drugs that successfully target those pathways in fragile X syndrome could potentially be re-purposed [127]. Other studies have used mesenchymal stem cells [163] or pharmacological treatment to block or antagonize the effect of MIA on purinergic [164] or inflammatory pathways [142, 165, 166].

## Future directions: ASD Living Biology

Evidence shows that ASD begins during prenatal life, which is the least studied and understood of all developmental periods of ASD. This glaring gap poses a major barrier to progress in ASD research and treatment and  exists because nearly all current approaches—postmortem, molecular, genomic, genetic, computational and animal models—cannot get answers to foundational questions such as: What are the underlying molecular and cellular processes during fetal and early post-natal stages that cause ASD in each child, and how does this vary across children? How do different prenatal trajectories explain and predict heterogeneity in post-natal brain growth and functioning, behavior and clinical profiles in ASD toddlers? Are there subtypes that display normal fetal-stage development but begin abnormal development at post-natal stages?

Nor can current approaches effectively examine more targeted questions such as: How do different *hcASD* genes affect fetal processes, and how are those effects related to post-natal cognitive, language and social development? Is there a difference in the brain development trajectory between idiopathic ASD cases and ASD with mutations in *hcASD* risk genes? Since macrocephaly is more strongly related to ASD than to intellectual disability (ID) [62] and many *hcASD* genes such as *CHD8* and *WDFY3* cause dysregulation of proliferation and brain overgrowth [193, 177, 183], what other fetal mechanisms are altered by excess proliferation that lead to an ASD outcome? What are the fetal-stage processes that alter imbalance between excitation and inhibition that is proposed to explain learning, memory, sensorimotor, and cognitive deficits in ASD [167]?

Post-natal phenotype data alone cannot reveal fetal-stage biology, and fetal-stage cellular models alone cannot explain post-natal development and clinical heterogeneity. There is a need to bridge the gap between fetal and post-natal biology for living ASD toddlers, and this requires a paradigm shift. This paradigm we describe here, ASD Living Biology, approaches the fundamental questions above, by acquiring fetal and early post-natal measures using iPS cells derived from skin or blood at any age, and integrating them using a within-subjects design. Discovery of the subtypes of prenatal pathogenic processes and their interplay with post-natal experiences should be a major direction for ASD research.

The ASD Living Biology approach will enable the construction of quantitative multiscale models that could address the perturbations in fetal-to-post-natal development. These explanatory and predictive models of ASD could address the disorder at the individual, subtype and group level, when compared to models from typically developing children. ASD Living Biology models would link in vitro and in vivo data. In vitro data would include molecular, cellular and physiological measures of a child’s iPS cells, including measures of fetal-stage proliferation/differentiation, cell fate and growth, synaptogenesis, synaptic function and synchronized neural network activity. This iPSC based approach could then be connected with in vivo measurements from the same child, including their neural structural and functional, genetic and genomic, behavioral, psychometric, diagnostic and clinical outcome data. By thus integrating such within-child fetal and post-natal measures, and integrating these data, it will be possible to better understand prenatal molecular mechanisms leading to the development of ASD.

### Interactions of post-natal biology and experiences with preceding prenatal biology

Future research could also determine whether some ASD prenatal trajectories are more deterministic of outcome phenotype, while others might be easily modified by experience, learning and interventions. The pleiotropic effect of many *hcASD* genes raises the testable hypothesis of a layered prenatal to post-natal path toward ASD in which the disorder may be dynamically impacted by post-natal changing functional roles of some ASD genes. It is also possible that heterogeneity in ASD outcomes may derive from the interplay of diverse prenatal dysregulations with post-natal exposures. Post-natal changes are thought to be reflected in changing behavioral and neurodevelopmental features [168] and some underlying mechanisms have been proposed [169]. Perhaps different prenatal pathogenic subtypes act as different general starting points from which diverse post-natal experiences lead to further variation in atypical development, some to higher and others to lower risk for ASD. For each child, prenatal starting points must be “worked around” to find optimal adaptations for that individual. As a result, more or less severe atypical behavior and cognitive outcomes may occur [170, 171]. ASD Living Biology studies could be designed to look at the interaction between atypical prenatal biology and post-natal experience-based adaptation and how this could lead to a spiral in early development that canalizes [172] over time toward multiple, heterogeneous end points (e.g., multifinality) [173]. Because neural circuits are sculpted by experience, future designs could examine whether atypical reduced social visual engagements from very early in life in ASD [174–176], are the product of some atypical prenatal to post-natal neural pathophysiology that causes an individual to engage in behavioral and cognitive adaptations that result in decreased sampling of the important social information necessary for typical social brain development. The more atypical such post-natal experience becomes, the more an individual’s development could become canalized for atypical outcomes.

## Conclusions

**Fig. 8:**
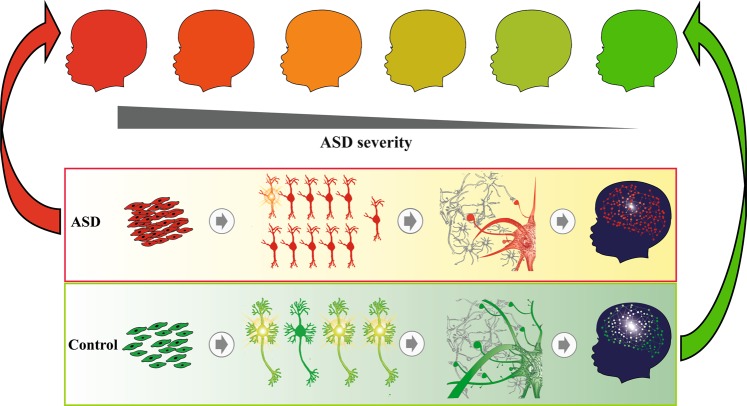
ASD is a multistage, progressive disorder of prenatal brain development. ASD children show a continuum of disorder severity because a wide range of heterogeneous insults can affect brain development in a not fully deterministic way. We propose the general theory that ASD arises from disruption of gene regulatory circuits with multiscale, hierarchical consequences on brain development starting from very early fetal stages. One possible prenatal trajectory of ASD is illustrated here. As compared with four fetal stages illustrated in typical development (lower panel; increasing fetal age from left to right), this ASD trajectory begins in the 1st and 2nd trimesters with abnormally high rates of proliferation; this results in excess neural precursor cells (ASD first panel). Disorder continues with disruption of migration, laminar disorganization, reduced cell growth, and reduced neurite outgrowth, resulting in neurons with a 10-fold decrease in spontaneous neural activity (ASD second panel). At still later stages, ASD neurons show defects in synaptogenesis, receptor and neurotransmitter development (ASD third panel). This deviant development of neurons leads to abnormal neural circuitry with a 6-fold decrease in synchronized bursts of neural network activity (ASD fourth panel). This also illustrates that ASD pathogenesis is not set at one point in time and does not reside in one process, but rather is a cascade of pathogenic processes. Different causes and prenatal times of insult combined with individual-dependent background genetics may alter details of developmental trajectories, resulting in differences in number, size and type of neurons in different cortical layers as well as number and functionality of synapses. This fetal heterogeneity leads to post-natal heterogeneity in neural circuits, behavior and clinical outcomes. Discovery of prenatal causes, processes and trajectories as they occur in children with ASD requires a paradigm shift: the ASD Living Biology approach. Courtesy of Eric Courchesne and Vahid H. Gazestani

Multiple lines of postmortem, cellular, molecular, genomic, genetic and animal model studies show that altered development in ASD can begin as early as the 1st and 2nd trimesters. Ninety-four percent of *hcASD* genes have peak expression prenatally, with the vast majority affecting proliferation/differentiation, cell fate, migration, neurite outgrowth and synaptogenesis. The majority are also pleiotropic, affecting multiple developmental stages, not just one. A commonly implicated pathway, RAS/ERK-PI3K/AKT, is also pleiotropic and impacts multiple prenatal stages from proliferation to synapse formation and function. In different ASD individuals, variation in how and when these pleiotropic pathways are dysregulated will lead to different, even opposing effects, producing prenatal as well as later neural and clinical heterogeneity. Thus, an excess or reduction of synapse formation may occur in different ASD cases; excess brain growth occurs in most while reduced size occurs in a small subset. Nonetheless, in each individual ASD child, multiple fetal-stage processes are disrupted; one pathogenesis pattern is illustrated in Fig. 8 and is based on Marchetto et al. [54]. Thus, the pathogenesis of ASD is not set at one point in time and does not reside in one process, but rather is a cascade of pathogenic processes. This cascade can begin as early as the first trimester, with alterations in cell proliferation and differentiation. The process then continues throughout prenatal life and into the early post-natal period of life. Affected regions include frontal, temporal, parietal, occipital, cerebellar, striatal, amygdala and hippocampal regions (Fig. 4). Remarkably, one ASD child-based cellular model finds a 10-fold decrease in spontaneous neural activity and a 6-fold decrease in synchronized bursts of neural network activity, a type of defect theorized long ago [6]. This dysfunctional network endpoint is not the “cause” of ASD but rather appears to stand at the end of a long chain of prenatal and early post-natal pathogenic changes (Fig. 8).

ASD is a multi-etiology, multi-stage, progressive disorder that spans most of fetal life. In our theory, a single perturbation can trigger a cascade of pathogenic changes, such as an MIA event, a single fetal injection of XAV939 [112] or *PTEN* mutation in a child [54]. Multiple perturbations may substantially amplify both the severity of disorder and outcome heterogeneity, such as, for example, a combination of *PTEN* mutation and MIA [130]. Moreover, many known and proposed ASD etiologies (e.g., *MECP2, PTEN*, MIA, XAV939) may perturb the pleiotropic RAS/ERK-PI3K/AKT pathway (Fig. 6), which in turn modulates each of the ASD-critical fetal and early post-natal stages discussed here. Dysregulated proliferation/differentiation can be a potent perturbation because it can lead to abnormal changes in subsequent stages of cell fate, migration, organization, maturation, and synapse and neural development [54, 112]. Cell and animal models of high-confidence ASD genes, such as *CHD8, PTEN, WDFY3*, definitively show disruption of cell proliferation (Fig. 5; Table S2); Fang et al. show robust synaptic, neural and behavioral downstream effects of excess neuron proliferation [112], and ASD patient-derived iPSC models show that excess proliferation is a robust early fetal stage defect in every case examined [54, 55].

Culprit prenatal perturbations do not necessarily produce identical neural and clinical outcomes even when the perturbations are themselves identical in siblings. The Choi and colleagues [132, 81] MIA ASD mouse model study showed that the same fetal MIA perturbation of littermates causes patches of focal cortical dysplasia that have marked heterogeneity, analogous to heterogeneity found in cortex in unrelated individual ASD cases postmortem [34] (Fig. 7). The *Wdfy3* mutation mouse model of ASD likewise displays heterogeneity of migration and focal patch defects across individual animals, as well as disrupted proliferation/differentiation and cortical overgrowth [177]. In Courchesne et al. [24], two ASD individuals had >100% the normal mean prefrontal neuron number, but one had small neurons and a modestly enlarged brain weight while the other had typical neuron size and one of the largest brain weights ever recorded for ASD. In the Marchetto et al. [54] study, even though neural progenitors from every ASD toddler doubled cell numbers faster than every control, the proliferation rates among ASD toddlers varied and that variation correlated with variation in MRI volume.

Because ASD begins in prenatal and early post-natal life, there is an ethical demand for early detection, intervention and services. When it is not detected in an infant or toddler, it is because it was missed [178]. Studies demonstrate that ASD risk can be measured as young as 12 to 24 months using parent report screening tools such as the CSBS IT-Checklist and M-CHAT at well-baby check-ups with follow-up confirmation at ASD specialty clinics [179, 180]. This screening procedure is fast, easy, inexpensive and effective, and can be done in any pediatric office or clinic [179]. Studies show that when pediatricians and ASD specialty clinics work together, risk detection and diagnostic evaluation can occur as early as 12 to 20 months, and interventions soon thereafter [179, 181].

Early interventions and services may improve an ASD child’s developmental outcome and help parents at a crucial time in human brain development. During the first post-natal years, the human brain undergoes a profound period of establishing and refining neural connections, and this is the basis for the emergence of higher-order social, language and cognitive networks and behavior. This important developmental step of the construction of functional and adaptive neural circuits is dependent on adaptive neural responses to input from the environment. If an infant or toddler with ASD is identified and behavioral treatment begun before or while early brain connections are being actively established, then brain function for that toddler stands the best chance of being improved. This is superior to treatment that begins late and after abnormal mature circuitry is already established. It is for this very reason that the early identification and treatment of ASD is essential and ethically demanded [181].

Lastly, to address the progressive prenatal beginnings and likely prenatal to post-natal experience-dependent interactions in ASD, new treatment designs are needed. We advocate coordinated in vitro and in vivo within-subject ASD Living Biology research designs to provide early-age molecular, cellular, and neuronal functional developmental explanations for individual differences as well as across ASD commonalities. Advances in stem cell model systems enable this [182]: Patient-derived brain organoids allow investigation of fetal processes that are remarkably similar to in vivo development. Within-child in vitro and in vivo designs could reveal prenatal and early post-natal bases of outcome clinical heterogeneity. Through this ASD Living Biology approach, relationships between prenatal development and post-natal experience, learning, brain growth and function, clinical presentation and progression, and treatment responsiveness may be defined at the individual level, thus enabling the development of precision medicine approaches with truly beneficial interventions.

## Electronic supplementary material

Supplementary Table S1

Supplementary Table S2

Supplementary Table S3

Supplementary Table S4
