# Supplementary material for: The ASD Living Biology: from cell proliferation to clinical phenotype
Source: Mol Psychiatry. 2018 Jun 22;24(1):88–107. doi: 10.1038/s41380-018-0056-y (PMC6309606; doi:10.1038/s41380-018-0056-y)
Supplement: Supplementary file 1 — Supplementary Table S1 [file 41380_2018_56_MOESM1_ESM.pdf]

V1	V2	V3	V4	V5	V6	V7	V8	V9	V20	V21	V38	V39
1	425-02	1	1	1	4	30	4560	1,160	20	204.0	6306351188	70916741
3	443-02	1	2	1	5	13	1568	1,390	21	212.7	3611899688	66564929
5	M5-03	1	1	2	8	22	196	1,570	21	247.7	3403831641	71340755
7	427-02	1	2	1	11	13	311	1,460	20	211.8	5961559500	82030000
8	445-02	1	1	1	13	8	75	1,470	20	257.7	4333685484	60344897
2	15-763-95	2	1	1	4	3	67	1,380	19	279.2	4357430375	55334552
4	426-02	2	2	2	4	21	233	1,222	20	191.8	3209247615	83320000
6	15-138-97	2	2	2	7	74	1290	1,350	20	212.9	3167730719	114097319
9	M9-03	2	1	2	14	20	1067	1,464	21	240.8	3148859162	60615474

LIST OF VARIABLES

V1 Brain number (1, 2, 3…)
V2 Code
V3 Diagnosis, A = 1; C = 2
V4 Sex, M = 1; F = 2
V5 Hemisphere, L = 1; R = 2
V6 Age (y)
V7 PMI (h)
V8 Fixation time (storage days) (d)
V9 Brain weight (g)
V20 Number of sections analyzed for volumes and total neuron numbers of the entire hemisphere
V21 Volume of the whole hemisphere (comprising white matter, cortical gray matter & subcortical gray matter) [cm3] (calculated considering the actual section thickness after histological processing)
V38 Total neuron number in the entire cortical gray
V39 Total neuron number in area 17

V38
6306351188
3611899688
3403831641
5961559500
4333685484
4357430375
3209247615
3167730719
3148859162

V39
70916741
66564929
71340755
82030000
60344897
55334552
83320000
114097319
60615474
