# Supplementary material for: The ASD Living Biology: from cell proliferation to clinical phenotype
Source: Mol Psychiatry. 2018 Jun 22;24(1):88–107. doi: 10.1038/s41380-018-0056-y (PMC6309606; doi:10.1038/s41380-018-0056-y)
Supplement: Supplementary file 2 — Supplementary Table S2 [file 41380_2018_56_MOESM2_ESM.pdf]

Table S2

Functional categories of hcASD genes. This spreadsheet is the master file of Figure 5 and includes the references used to identify the functional roles of hcASD genes during brain development.

CLUSTER	GENE NAME	PROLIFERATION	NEURON MIGRATION	NEURITE GROWTH NEURONPROJECTION	SYNAPSE FUNCTION/ASSEMBLY
Green	TBR1		[1]		
Purple	FOXP1		[2,3]	[3]	
Purple	MBOAT7		[4]	[4]	
Green	NCKAP1		[5]	[5]	
Green	ANKRD11	[6]	[6]		
Green	KDM5B	[7,8]	[7] (adult neural stem cells)		
Green	BCL11A		[9]		
Green	CUL3	[10] (not neural cell); [11-13]	[13,14]	[13,15]	
Green	CNTNAP2		[16]	inferential: [17,18]	[19]
Green	RELN		[20]	[21]	[22]
Green	WDFY3	[23]	[23]	[24]	[24]
Green	PTEN	[25]	[26]	[27,28]	[29]
Green	MECP2	[30]	[30,31]	[32]	[33,34]
Purple	KAT2B	inferential: [35]	[36]	[37](shown to increase axonal regeneration)	[36]
Purple	MET	oligodendrocyte: [38]	[39]	[40]	[40]
Green	ASXL3				
Purple	BCKDK				
Purple	GALNT18				
Green	gigyf1				
Green	GIGYF2				
Green	ILF2				
Green	IRF2BPL				
Purple	KATNAL 2				
Green	KMT2C				
Green	MED13L				
Green	PHF2				
Green	RANBP17				
Green	TNRC6B				
Green	ZC3H4				
Green	DIP2A			[41-43]	
Purple	CNTN4			[44,45]	
N/A	MAGEL2			[46,47]	
N/A	MSNP1AS			[48]	
Purple	TCF7L2	[49]			
N/A	CHD2	[50]			
Green	MYT1L	[51]			

Green	KMT5B	[52](adult neural stem cells)			
Green	KMT5C	[52](adult neural stem cells)			
Green	TRIP12	embryonic stem cells: [53]			
Green	KMT2E	inferential: [54]			
Green	WAC	inferential: [55,56]			
Green	SETD5	[57](embryonic stem cells)			
Purple	RIMS1				[58-60]
Green	GABRB3				[61]
Green	GRIN2B				[62]
Purple	PTCHD1				[63]
Purple	SCN2A				[64]
Purple	SHANK2				[65]
Purple	SLC6A1				[61]
Green	POGZ	[66]		[67]	
Green	INTS6	[68](from breast cancer)		[69](complex involved in migration)	
Green	SPAST	[70,71]		[72,73]	
Purple	SYNGAP1			[74]	[74,75]
Purple	ANK2			[76]	[77]
Green	CACNA1H			[78]	[78]
Green	DSCAM			[79,80]	[81]
Green	GRIP1			[82]	[83]
Purple	NRXN1			[84]	[85]
Green	USP7	[86]			[87]
Green	KDM6B	[88]			[89]
Green	KMT2A	[90]			[91]
Green	CHD8	[92,93]			[94]
Purple	CACNA2D3	[95,96]			[97]
Green	ASH1L	[98](hematopoietic stem cells)			[99]
Purple	DEAF1	inferential: [100,101]			[102]
Purple	ERBIN	neurons: [103]; Schwann cells:[104]			[105]
Green	DYRK1A	[106]		[107]	[108]
Green	ARID1B	[109]		[110 111]	[110]
Purple	NLGN3	[112]		[113]	[114]
Purple	SHANK3	[115]		[116]	[117]
Green	CTNND2	glioma cells: [118]		[119,120]	[121]
Green	ADNP			[122]	[123]

SUPPLEMENTARY REFERENCES

1 Hevner, R. F. *et al.* Tbr1 regulates differentiation of the preplate and layer 6. *Neuron* **29**, 353-366 (2001).

2 Rousso, D. L., Gaber, Z. B., Wellik, D., Morrisey, E. E. & Novitch, B. G. Coordinated actions of the forkhead protein Foxp1 and Hox proteins in the columnar organization of spinal motor neurons. *Neuron* **59**, 226-240, doi:10.1016/j.neuron.2008.06.025 (2008).

3 Li, X. *et al.* Foxp1 regulates cortical radial migration and neuronal morphogenesis in developing cerebral cortex. *PLoS One* **10**, e0127671, doi:10.1371/journal.pone.0127671 (2015).

4 Lee, H. C. *et al.* LPIAT1 regulates arachidonic acid content in phosphatidylinositol and is required for cortical lamination in mice. *Mol Biol Cell* **23**, 4689-4700, doi:10.1091/mbc.E12-09-0673 (2012).

5 Yokota, Y., Ring, C., Cheung, R., Pevny, L. & Anton, E. S. Nap1-regulated neuronal cytoskeletal dynamics is essential for the final differentiation of neurons in cerebral cortex. *Neuron* **54**, 429-445, doi:10.1016/j.neuron.2007.04.016 (2007).

6 Gallagher, D. *et al.* Ankrd11 is a chromatin regulator involved in autism that is essential for neural development. *Dev Cell* **32**, 31-42, doi:10.1016/j.devcel.2014.11.031 (2015).

7 Zhou, Q. *et al.* Inhibition of the histone demethylase Kdm5b promotes neurogenesis and derepresses Reln (reelin) in neural stem cells from the adult subventricular zone of mice. *Mol Biol Cell* **27**, 627-639, doi:10.1091/mbc.E15-07-0513 (2016).

8 Xie, L. *et al.* KDM5B regulates embryonic stem cell self-renewal and represses cryptic intragenic transcription. *EMBO J* **30**, 1473-1484, doi:10.1038/emboj.2011.91 (2011).

9 Wiegreffe, C. *et al.* Bcl11a (Ctip1) Controls Migration of Cortical Projection Neurons through Regulation of Sema3c. *Neuron* **87**, 311-325, doi:10.1016/j.neuron.2015.06.023 (2015).

10 Singer, J. D., Gurian-West, M., Clurman, B. & Roberts, J. M. Cullin-3 targets cyclin E for ubiquitination and controls S phase in mammalian cells. *Genes Dev* **13**, 2375-2387 (1999).

11 Zhu, S., Korzh, V., Gong, Z. & Low, B. C. RhoA prevents apoptosis during zebrafish embryogenesis through activation of Mek/Erk pathway. *Oncogene* **27**, 1580-1589, doi:10.1038/sj.onc.1210790 (2008).

12 Chen, L., Melendez, J., Campbell, K., Kuan, C. Y. & Zheng, Y. Rac1 deficiency in the forebrain results in neural progenitor reduction and microcephaly. *Dev Biol* **325**, 162-170, doi:10.1016/j.ydbio.2008.10.023 (2009).

13 Lin, G. N. *et al.* Spatiotemporal 16p11.2 protein network implicates cortical late mid-fetal brain development and KCTD13-Cul3-RhoA pathway in psychiatric diseases. *Neuron* **85**, 742-754, doi:10.1016/j.neuron.2015.01.010 (2015).

14 Chen, Y. *et al.* Cullin mediates degradation of RhoA through evolutionarily conserved BTB adaptors to control actin cytoskeleton structure and cell movement. *Mol Cell* **35**, 841-855, doi:10.1016/j.molcel.2009.09.004 (2009).

15 Kasahara, K. *et al.* Ubiquitin-proteasome system controls ciliogenesis at the initial step of axoneme extension. *Nat Commun* **5**, 5081, doi:10.1038/ncomms6081 (2014).

16 Penagarikano, O. *et al.* Absence of CNTNAP2 leads to epilepsy, neuronal migration abnormalities, and core autism-related deficits. *Cell* **147**, 235-246, doi:10.1016/j.cell.2011.08.040 (2011).

17 Vernes, S. C. *et al.* Foxp2 regulates gene networks implicated in neurite outgrowth in the developing brain. *PLoS Genet* **7**, e1002145, doi:10.1371/journal.pgen.1002145 (2011).

18 Vernes, S. C. *et al.* A functional genetic link between distinct developmental language disorders. *N Engl J Med* **359**, 2337-2345, doi:10.1056/NEJMoa0802828 (2008).

19 Gdalyahu, A. *et al.* The Autism Related Protein Contactin-Associated Protein-Like 2 (CNTNAP2) Stabilizes New Spines: An In Vivo Mouse Study. *PLoS One* **10**, e0125633, doi:10.1371/journal.pone.0125633 (2015).

20 Baek, S. T. *et al.* An AKT3-FOXG1-reelin network underlies defective migration in human focal malformations of cortical development. *Nat Med* **21**, 1445-1454, doi:10.1038/nm.3982 (2015).

21 Barros, C. S., Franco, S. J. & Muller, U. Extracellular matrix: functions in the nervous system. *Cold Spring Harb Perspect Biol* **3**, a005108, doi:10.1101/cshperspect.a005108 (2011).

22 Herz, J. & Chen, Y. Reelin, lipoprotein receptors and synaptic plasticity. *Nat Rev Neurosci* **7**, 850-859, doi:10.1038/nrn2009 (2006).

23 Orosco, L. A. *et al.* Loss of Wdfy3 in mice alters cerebral cortical neurogenesis reflecting aspects of the autism pathology. *Nat Commun* **5**, 4692, doi:10.1038/ncomms5692 (2014).

24 Dragich, J. M. *et al.* Autophagy linked FYVE (Alfy/WDFY3) is required for establishing neuronal connectivity in the mammalian brain. *Elife* **5**, doi:10.7554/eLife.14810 (2016).

25 Chen, Y., Huang, W. C., Sejourne, J., Clipperton-Allen, A. E. & Page, D. T. Pten Mutations Alter Brain Growth Trajectory and Allocation of Cell Types through Elevated beta-Catenin Signaling. *J Neurosci* **35**, 10252-10267, doi:10.1523/JNEUROSCI.5272-14.2015 (2015).

26 Li, L. *et al.* PTEN in neural precursor cells: regulation of migration, apoptosis, and proliferation. *Mol Cell Neurosci* **20**, 21-29, doi:10.1006/mcne.2002.1115 (2002).

27 Park, K. K. *et al.* Promoting axon regeneration in the adult CNS by modulation of the PTEN/mTOR pathway. *Science* **322**, 963-966, doi:10.1126/science.1161566 (2008).

28 Kwon, C. H. *et al.* Pten regulates neuronal arborization and social interaction in mice. *Neuron* **50**, 377-388, doi:10.1016/j.neuron.2006.03.023 (2006).

29 Takeuchi, K. *et al.* Dysregulation of synaptic plasticity precedes appearance of morphological defects in a Pten conditional knockout mouse model of autism. *Proc Natl Acad Sci U S A* **110**, 4738-4743, doi:10.1073/pnas.1222803110 (2013).

30 Mellios, N. *et al.* MeCP2-regulated miRNAs control early human neurogenesis through differential effects on ERK and AKT signaling. *Mol Psychiatry*, doi:10.1038/mp.2017.86 (2017).

31 Zhang, Z. N. *et al.* Layered hydrogels accelerate iPSC-derived neuronal maturation and reveal migration defects caused by MeCP2 dysfunction. *Proc Natl Acad Sci U S A* **113**, 3185-3190, doi:10.1073/pnas.1521255113 (2016).

32 Cheng, T. L. *et al.* MeCP2 suppresses nuclear microRNA processing and dendritic growth by regulating the DGCR8/Drosha complex. *Dev Cell* **28**, 547-560, doi:10.1016/j.devcel.2014.01.032 (2014).

33 Marchetto, M. C. *et al.* A model for neural development and treatment of Rett syndrome using human induced pluripotent stem cells. *Cell* **143**, 527-539, doi:10.1016/j.cell.2010.10.016 (2010).

34 Li, Y. *et al.* Global transcriptional and translational repression in human-embryonic-stem-cell-derived Rett syndrome neurons. *Cell Stem Cell* **13**, 446-458, doi:10.1016/j.stem.2013.09.001 (2013).

35 Fournier, M. *et al.* KAT2A/KAT2B-targeted acetylome reveals a role for PLK4 acetylation in preventing centrosome amplification. *Nat Commun* **7**, 13227, doi:10.1038/ncomms13227 (2016).

36 Maurice, T. *et al.* Altered memory capacities and response to stress in p300/CBP-associated factor (PCAF) histone acetylase knockout mice. *Neuropsychopharmacology* **33**, 1584-1602, doi:10.1038/sj.npp.1301551 (2008).

37 Puttagunta, R. *et al.* PCAF-dependent epigenetic changes promote axonal regeneration in the central nervous system. *Nat Commun* **5**, 3527, doi:10.1038/ncomms4527 (2014).

38 Ohya, W., Funakoshi, H., Kurosawa, T. & Nakamura, T. Hepatocyte growth factor (HGF) promotes oligodendrocyte progenitor cell proliferation and inhibits its differentiation during postnatal development in the rat. *Brain Res* **1147**, 51-65, doi:10.1016/j.brainres.2007.02.045 (2007).

39 Powell, E. M., Mars, W. M. & Levitt, P. Hepatocyte growth factor/scatter factor is a motogen for interneurons migrating from the ventral to dorsal telencephalon. *Neuron* **30**, 79-89 (2001).

40 Peng, Y. *et al.* The autism-associated MET receptor tyrosine kinase engages early neuronal growth mechanism and controls glutamatergic circuits development in the forebrain. *Mol Psychiatry* **21**, 925-935, doi:10.1038/mp.2015.182 (2016).
41 Mukhopadhyay, M. *et al.* Cloning, genomic organization and expression pattern of a novel Drosophila gene, the disco-interacting protein 2 (dip2), and its murine homolog. *Gene* **293**, 59-65 (2002).
42 Liang, X. *et al.* Follistatin-like 1 attenuates apoptosis via disco-interacting protein 2 homolog A/Akt pathway after middle cerebral artery occlusion in rats. *Stroke* **45**, 3048-3054, doi:10.1161/STROKEAHA.114.006092 (2014).
43 Nitta, Y., Yamazaki, D., Sugie, A., Hiroi, M. & Tabata, T. DISCO Interacting Protein 2 regulates axonal bifurcation and guidance of Drosophila mushroom body neurons. *Dev Biol* **421**, 233-244, doi:10.1016/j.ydbio.2016.11.015 (2017).
44 Bray, N. Axon guidance: Setting up for seeing in slow motion. *Nat Rev Neurosci* **16**, 374-375, doi:10.1038/nrn3979 (2015).
45 Mercati, O. *et al.* Contactin 4, -5 and -6 differentially regulate neuritogenesis while they display identical PTPRG binding sites. *Biol Open* **2**, 324-334, doi:10.1242/bio.20133343 (2013).
46 Lee, S. *et al.* Essential role for the Prader-Willi syndrome protein necdin in axonal outgrowth. *Hum Mol Genet* **14**, 627-637, doi:10.1093/hmg/ddi059 (2005).
47 Maillard, J. *et al.* Loss of Magel2 impairs the development of hypothalamic Anorexigenic circuits. *Hum Mol Genet* **25**, 3208-3215, doi:10.1093/hmg/ddw169 (2016).
48 DeWitt, J. J. *et al.* Impact of the Autism-Associated Long Noncoding RNA MSNP1AS on Neuronal Architecture and Gene Expression in Human Neural Progenitor Cells. *Genes (Basel)* **7**, doi:10.3390/genes7100076 (2016).
49 Nazwar, T. A., Glassmann, A. & Schilling, K. Expression and molecular diversity of Tcf7l2 in the developing murine cerebellum and brain. *J Neurosci Res* **87**, 1532-1546, doi:10.1002/jnr.21989 (2009).
50 Shen, T., Ji, F., Yuan, Z. & Jiao, J. CHD2 is Required for Embryonic Neurogenesis in the Developing Cerebral Cortex. *Stem Cells* **33**, 1794-1806, doi:10.1002/stem.2001 (2015).
51 Mall, M. *et al.* Myt1l safeguards neuronal identity by actively repressing many non-neuronal fates. *Nature* **544**, 245-249, doi:10.1038/nature21722 (2017).
52 Rhodes, C. T. *et al.* Cross-species Analyses Unravel the Complexity of H3K27me3 and H4K20me3 in the Context of Neural Stem Progenitor Cells. *Neuroepigenetics* **6**, 10-25, doi:10.1016/j.nepig.2016.04.001 (2016).
53 Kajiro, M. *et al.* The E3 ubiquitin ligase activity of Trip12 is essential for mouse embryogenesis. *PLoS One* **6**, e25871, doi:10.1371/journal.pone.0025871 (2011).
54 Wynder, C., Hakimi, M. A., Epstein, J. A., Shilatifard, A. & Shiekhattar, R. Recruitment of MLL by HMG-domain protein iBRAF promotes neural differentiation. *Nat Cell Biol* **7**, 1113-1117, doi:10.1038/ncb1312 (2005).
55 David-Morrison, G. *et al.* WAC Regulates mTOR Activity by Acting as an Adaptor for the TTT and Pontin/Reptin Complexes. *Dev Cell* **36**, 139-151, doi:10.1016/j.devcel.2015.12.019 (2016).
56 Zhang, F. & Yu, X. WAC, a functional partner of RNF20/40, regulates histone H2B ubiquitination and gene transcription. *Mol Cell* **41**, 384-397, doi:10.1016/j.molcel.2011.01.024 (2011).
57 Osipovich, A. B., Gangula, R., Vianna, P. G. & Magnuson, M. A. Setd5 is essential for mammalian development and the co-transcriptional regulation of histone acetylation. *Development* **143**, 4595-4607, doi:10.1242/dev.141465 (2016).
58 Branco, T. & Staras, K. The probability of neurotransmitter release: variability and feedback control at single synapses. *Nat Rev Neurosci* **10**, 373-383, doi:10.1038/nrn2634 (2009).
59 Betz, A. *et al.* Functional interaction of the active zone proteins Munc13-1 and RIM1 in synaptic vesicle priming. *Neuron* **30**, 183-196 (2001).
60 Camacho, M. *et al.* Heterodimerization of Munc13 C2A domain with RIM regulates synaptic vesicle docking and priming. *Nat Commun* **8**, 15293, doi:10.1038/ncomms15293 (2017).

61 Glykys, J. & Mody, I. Activation of GABAA receptors: views from outside the synaptic cleft. *Neuron* **56**, 763-770, doi:10.1016/j.neuron.2007.11.002 (2007).
62 Endele, S. *et al.* Mutations in GRIN2A and GRIN2B encoding regulatory subunits of NMDA receptors cause variable neurodevelopmental phenotypes. *Nat Genet* **42**, 1021-1026, doi:10.1038/ng.677 (2010).
63 Ung, D. C. *et al.* Ptchd1 deficiency induces excitatory synaptic and cognitive dysfunctions in mouse. *Mol Psychiatry*, doi:10.1038/mp.2017.39 (2017).
64 Ben-Shalom, R. *et al.* Opposing Effects on NaV1.2 Function Underlie Differences Between SCN2A Variants Observed in Individuals With Autism Spectrum Disorder or Infantile Seizures. *Biol Psychiatry* **82**, 224-232, doi:10.1016/j.biopsych.2017.01.009 (2017).
65 Won, H. *et al.* Autistic-like social behaviour in Shank2-mutant mice improved by restoring NMDA receptor function. *Nature* **486**, 261-265, doi:10.1038/nature11208 (2012).
66 Fukai, R. *et al.* A case of autism spectrum disorder arising from a de novo missense mutation in POGZ. *J Hum Genet* **60**, 277-279, doi:10.1038/jhg.2015.13 (2015).
67 Hashimoto, R. *et al.* Whole-exome sequencing and neurite outgrowth analysis in autism spectrum disorder. *J Hum Genet* **61**, 199-206, doi:10.1038/jhg.2015.141 (2016).
68 Wieland, I., Sell, C., Weidle, U. H. & Wieacker, P. Ectopic expression of DICE1 suppresses tumor cell growth. *Oncol Rep* **12**, 207-211 (2004).
69 van den Berg, D. L. *et al.* Nipbl Interacts with Zfp609 and the Integrator Complex to Regulate Cortical Neuron Migration. *Neuron* **93**, 348-361, doi:10.1016/j.neuron.2016.11.047 (2017).
70 Connell, J. W., Lindon, C., Luzio, J. P. & Reid, E. Spastin couples microtubule severing to membrane traffic in completion of cytokinesis and secretion. *Traffic* **10**, 42-56, doi:10.1111/j.1600-0854.2008.00847.x (2009).
71 Vietri, M. *et al.* Spastin and ESCRT-III coordinate mitotic spindle disassembly and nuclear envelope sealing. *Nature* **522**, 231-235, doi:10.1038/nature14408 (2015).
72 Evans, K. J., Gomes, E. R., Reisenweber, S. M., Gundersen, G. G. & Lauring, B. P. Linking axonal degeneration to microtubule remodeling by Spastin-mediated microtubule severing. *J Cell Biol* **168**, 599-606, doi:10.1083/jcb.200409058 (2005).
73 Zhang, C. *et al.* Role of spastin and protrudin in neurite outgrowth. *J Cell Biochem* **113**, 2296-2307, doi:10.1002/jcb.24100 (2012).
74 Berryer, M. H. *et al.* Decrease of SYNGAP1 in GABAergic cells impairs inhibitory synapse connectivity, synaptic inhibition and cognitive function. *Nat Commun* **7**, 13340, doi:10.1038/ncomms13340 (2016).
75 Komiyama, N. H. *et al.* SynGAP regulates ERK/MAPK signaling, synaptic plasticity, and learning in the complex with postsynaptic density 95 and NMDA receptor. *J Neurosci* **22**, 9721-9732 (2002).
76 Lorenzo, D. N. *et al.* A PIK3C3-ankyrin-B-dynactin pathway promotes axonal growth and multiorganelle transport. *J Cell Biol* **207**, 735-752, doi:10.1083/jcb.201407063 (2014).
77 Koch, I. *et al.* Drosophila ankyrin 2 is required for synaptic stability. *Neuron* **58**, 210-222, doi:10.1016/j.neuron.2008.03.019 (2008).
78 Eckle, V. S. *et al.* Mechanisms by which a CACNA1H mutation in epilepsy patients increases seizure susceptibility. *J Physiol* **592**, 795-809, doi:10.1113/jphysiol.2013.264176 (2014).
79 Ly, A. *et al.* DSCAM is a netrin receptor that collaborates with DCC in mediating turning responses to netrin-1. *Cell* **133**, 1241-1254, doi:10.1016/j.cell.2008.05.030 (2008).
80 Hattori, D. *et al.* Dscam diversity is essential for neuronal wiring and self-recognition. *Nature* **449**, 223-227, doi:10.1038/nature06099 (2007).
81 Cvetkovska, V., Hibbert, A. D., Emran, F. & Chen, B. E. Overexpression of Down syndrome cell adhesion molecule impairs precise synaptic targeting. *Nat Neurosci* **16**, 677-682, doi:10.1038/nn.3396 (2013).
82 Hoogenraad, C. C., Milstein, A. D., Ethell, I. M., Henkemeyer, M. & Sheng, M. GRIP1 controls dendrite morphogenesis by regulating EphB receptor trafficking. *Nat Neurosci* **8**, 906-915, doi:10.1038/nn1487 (2005).

83 Tan, H. L., Queenan, B. N. & Huganir, R. L. GRIP1 is required for homeostatic regulation of AMPAR trafficking. *Proc Natl Acad Sci U S A* **112**, 10026-10031, doi:10.1073/pnas.1512786112 (2015).
84 Gjorlund, M. D. *et al.* Neuroligin-1 induces neurite outgrowth through interaction with neurexin-1beta and activation of fibroblast growth factor receptor-1. *FASEB J* **26**, 4174-4186, doi:10.1096/fj.11-202242 (2012).
85 Maro, G. S. *et al.* MADD-4/Punctin and Neurexin Organize C. elegans GABAergic Postsynapses through Neuroligin. *Neuron* **86**, 1420-1432, doi:10.1016/j.neuron.2015.05.015 (2015).
86 Huang, Z. *et al.* Deubiquitylase HAUSP stabilizes REST and promotes maintenance of neural progenitor cells. *Nat Cell Biol* **13**, 142-152, doi:10.1038/ncb2153 (2011).
87 Tai, H. C., Besche, H., Goldberg, A. L. & Schuman, E. M. Characterization of the Brain 26S Proteasome and its Interacting Proteins. *Front Mol Neurosci* **3**, doi:10.3389/fnmol.2010.00012 (2010).
88 Li, W. *et al.* DNA methyltransferase mediates dose-dependent stimulation of neural stem cell proliferation by folate. *J Nutr Biochem* **24**, 1295-1301, doi:10.1016/j.jnutbio.2012.11.001 (2013).
89 Wijayatunge, R. *et al.* The histone lysine demethylase Kdm6b is required for activity-dependent preconditioning of hippocampal neuronal survival. *Mol Cell Neurosci* **61**, 187-200, doi:10.1016/j.mcn.2014.06.008 (2014).
90 Huang, Y. C. *et al.* The epigenetic factor Kmt2a/Mll1 regulates neural progenitor proliferation and neuronal and glial differentiation. *Dev Neurobiol* **75**, 452-462, doi:10.1002/dneu.22235 (2015).
91 Jakovcevski, M. *et al.* Neuronal Kmt2a/Mll1 histone methyltransferase is essential for prefrontal synaptic plasticity and working memory. *J Neurosci* **35**, 5097-5108, doi:10.1523/JNEUROSCI.3004-14.2015 (2015).
92 Sugathan, A. *et al.* CHD8 regulates neurodevelopmental pathways associated with autism spectrum disorder in neural progenitors. *Proc Natl Acad Sci U S A* **111**, E4468-4477, doi:10.1073/pnas.1405266111 (2014).
93 Cotney, J. *et al.* The autism-associated chromatin modifier CHD8 regulates other autism risk genes during human neurodevelopment. *Nat Commun* **6**, 6404, doi:10.1038/ncomms7404 (2015).
94 Platt, R. J. *et al.* Chd8 Mutation Leads to Autistic-like Behaviors and Impaired Striatal Circuits. *Cell Rep* **19**, 335-350, doi:10.1016/j.celrep.2017.03.052 (2017).
95 Jin, Y. *et al.* CACNA2D3 is downregulated in gliomas and functions as a tumor suppressor. *Mol Carcinog* **56**, 945-959, doi:10.1002/mc.22548 (2017).
96 Sun, Y. M. *et al.* Distinct profiles of REST interactions with its target genes at different stages of neuronal development. *Mol Biol Cell* **16**, 5630-5638, doi:10.1091/mbc.E05-07-0687 (2005).
97 Villela, D., Suemoto, C. K., Pasqualucci, C. A., Grinberg, L. T. & Rosenberg, C. Do Copy Number Changes in CACNA2D2, CACNA2D3, and CACNA1D Constitute a Predisposing Risk Factor for Alzheimer's Disease? *Front Genet* **7**, 107, doi:10.3389/fgene.2016.00107 (2016).
98 Jones, M. *et al.* Ash1l controls quiescence and self-renewal potential in hematopoietic stem cells. *J Clin Invest* **125**, 2007-2020, doi:10.1172/JCI78124 (2015).
99 Zhu, T. *et al.* Histone methyltransferase Ash1L mediates activity-dependent repression of neurexin-1alpha. *Sci Rep* **6**, 26597, doi:10.1038/srep26597 (2016).
100 Manne, U. *et al.* Altered subcellular localization of suppressin, a novel inhibitor of cell-cycle entry, is an independent prognostic factor in colorectal adenocarcinomas. *Clin Cancer Res* **7**, 3495-3503 (2001).
101 Barker, H. E. *et al.* Deaf-1 regulates epithelial cell proliferation and side-branching in the mammary gland. *BMC Dev Biol* **8**, 94, doi:10.1186/1471-213X-8-94 (2008).
102 Czesak, M., Lemonde, S., Peterson, E. A., Rogaeva, A. & Albert, P. R. Cell-specific repressor or enhancer activities of Deaf-1 at a serotonin 1A receptor gene polymorphism. *J Neurosci* **26**, 1864-1871, doi:10.1523/JNEUROSCI.2643-05.2006 (2006).
103 Huang, Y. Z., Zang, M., Xiong, W. C., Luo, Z. & Mei, L. Erbin suppresses the MAP kinase pathway. *J Biol Chem* **278**, 1108-1114, doi:10.1074/jbc.M205413200 (2003).

104 Rangwala, R., Banine, F., Borg, J. P. & Sherman, L. S. Erbin regulates mitogen-activated protein (MAP) kinase activation and MAP kinase-dependent interactions between Merlin and adherens junction protein complexes in Schwann cells. *J Biol Chem* **280**, 11790-11797, doi:10.1074/jbc.M414154200 (2005).
105 Tao, Y. *et al.* Erbin interacts with TARP gamma-2 for surface expression of AMPA receptors in cortical interneurons. *Nat Neurosci* **16**, 290-299, doi:10.1038/nn.3320 (2013).
106 Yabut, O., Domogauer, J. & D'Arcangelo, G. Dyrk1A overexpression inhibits proliferation and induces premature neuronal differentiation of neural progenitor cells. *J Neurosci* **30**, 4004-4014, doi:10.1523/JNEUROSCI.4711-09.2010 (2010).
107 Gockler, N. *et al.* Harmine specifically inhibits protein kinase DYRK1A and interferes with neurite formation. *FEBS J* **276**, 6324-6337, doi:10.1111/j.1742-4658.2009.07346.x (2009).
108 Ahn, K. J. *et al.* DYRK1A BAC transgenic mice show altered synaptic plasticity with learning and memory defects. *Neurobiol Dis* **22**, 463-472, doi:10.1016/j.nbd.2005.12.006 (2006).
109 Sim, J. C. *et al.* Expanding the phenotypic spectrum of ARID1B-mediated disorders and identification of altered cell-cycle dynamics due to ARID1B haploinsufficiency. *Orphanet J Rare Dis* **9**, 43, doi:10.1186/1750-1172-9-43 (2014).
110 Ka, M., Chopra, D. A., Dravid, S. M. & Kim, W. Y. Essential Roles for ARID1B in Dendritic Arborization and Spine Morphology of Developing Pyramidal Neurons. *J Neurosci* **36**, 2723-2742, doi:10.1523/JNEUROSCI.2321-15.2016 (2016).
111 Ronan, J. L., Wu, W. & Crabtree, G. R. From neural development to cognition: unexpected roles for chromatin. *Nat Rev Genet* **14**, 347-359, doi:10.1038/nrg3413 (2013).
112 Venkatesh, H. S. *et al.* Neuronal Activity Promotes Glioma Growth through Neuroligin-3 Secretion. *Cell* **161**, 803-816, doi:10.1016/j.cell.2015.04.012 (2015).
113 McFadden, K. & Minshew, N. J. Evidence for dysregulation of axonal growth and guidance in the etiology of ASD. *Front Hum Neurosci* **7**, 671, doi:10.3389/fnhum.2013.00671 (2013).
114 Sudhof, T. C. Neuroligins and neurexins link synaptic function to cognitive disease. *Nature* **455**, 903-911, doi:10.1038/nature07456 (2008).
115 Zhao, H. *et al.* Altered neurogenesis and disrupted expression of synaptic proteins in prefrontal cortex of SHANK3-deficient non-human primate. *Cell Res*, doi:10.1038/cr.2017.95 (2017).
116 Sheng, M. & Kim, E. The Shank family of scaffold proteins. *J Cell Sci* **113 (Pt 11)**, 1851-1856 (2000).
117 Lee, J. *et al.* Shank3-mutant mice lacking exon 9 show altered excitation/inhibition balance, enhanced rearing, and spatial memory deficit. *Front Cell Neurosci* **9**, 94, doi:10.3389/fncel.2015.00094 (2015).
118 Schonberg, D. L., Bao, S. & Rich, J. N. Genomics informs glioblastoma biology. *Nat Genet* **45**, 1105-1107, doi:10.1038/ng.2775 (2013).
119 Abu-Elneel, K. *et al.* A delta-catenin signaling pathway leading to dendritic protrusions. *J Biol Chem* **283**, 32781-32791, doi:10.1074/jbc.M804688200 (2008).
120 Kosik, K. S., Donahue, C. P., Israely, I., Liu, X. & Ochiishi, T. Delta-catenin at the synaptic-adherens junction. *Trends Cell Biol* **15**, 172-178, doi:10.1016/j.tcb.2005.01.004 (2005).
121 Turner, T. N. *et al.* Loss of delta-catenin function in severe autism. *Nature* **520**, 51-56, doi:10.1038/nature14186 (2015).
122 Mandel, S., Spivak-Pohis, I. & Gozes, I. ADNP differential nucleus/cytoplasm localization in neurons suggests multiple roles in neuronal differentiation and maintenance. *J Mol Neurosci* **35**, 127-141, doi:10.1007/s12031-007-9013-y (2008).
123 Oz, S. *et al.* The NAP motif of activity-dependent neuroprotective protein (ADNP) regulates dendritic spines through microtubule end binding proteins. *Mol Psychiatry* **19**, 1115-1124, doi:10.1038/mp.2014.97 (2014).
