# Supplementary material for: The ASD Living Biology: from cell proliferation to clinical phenotype
Source: Mol Psychiatry. 2018 Jun 22;24(1):88–107. doi: 10.1038/s41380-018-0056-y (PMC6309606; doi:10.1038/s41380-018-0056-y)
Supplement: Supplementary file 3 — Supplementary Table S3 [file 41380_2018_56_MOESM3_ESM.pdf]

Table S3. Examples of hcASD genes that converge on and disrupt RAS/ERK, PI3K/AKT, Wnt and β-catenin signaling pathways.

Gene name	Pathway involved	Examples of Effects on Pathways
FMR1 ***b,c,d***	**PI3K/AKT**	*Fmr1* knock-out mice have elevated activation of PI3K/AKT signaling pathway, affecting synaptic plasticity with behavioral and cognitive defects[1]. Down-regulation of PI3K in prefrontal cortex of *Fmr1* null mice ameliorates aberrations in protein synthesis, dendritic spine density and cortical network activity[1,2].
FOXG1 ***b***	**PI3K/AKT**	The PI3K/AKT signaling pathway affects cell fate determination and neural migration by regulating *FOXG1*[3-5].
NLGN3 ***a,c,d***	**PI3K/AKT**	*NLGN3* secretion by active neurons enhances proliferation of glial cells by activation of PI3K/AKT pathway. Also affects synaptic development[6].
PTEN ***a,b,c,d***	**PI3K/AKT**	*Pten*$^{+/-}$ mice show high activity in PI3K/AKT and β-catenin signaling resulting in enhanced proliferation of radial glial cells and cortical overgrowth[7]; neuronal hypertrophy, hypertrophic and ectopic dendrites and axonal tracts[8]; increased synapses[8]; and hyperconnectivity of prefrontal cortex with amygdala[9]. *Pten* also involves in neural migration[10,11].
RELN ***b,c,d***	**PI3K/AKT**	PI3K/AKT signaling pathway affects cell fate determination and neural migration by regulating *RELN*[12]. While RELN promotes neuronal maturation, synaptic formation and plasticity, it appears to be more important for dendrite and spine development via PI3K/Akt/mTOR pathway.
SHANK3 ***a,c,d***	**PI3K/AKT**	Mice with *Shank3* mutations show deficits in synaptic function, hippocampal LTP and motor performance that can be improved with IGF1 treatment[13-15]. Also affects neurogenesis.
MECP2 ***a,b,c,d***	**PI3K/AKT** **RAS/ERK**	iPS-derived RG cells from Rett syndrome patients with *MECP2* mutation exhibit excess proliferation through activation of PI3K/AKT and down-regulation of RAS/ERK pathways[16]. Neurons with *MECP2* knock-down fail to attain a unipolar/bipolar shape, display abnormal migration and reduced cortical thickness[16]. Neuron models of *MECP2* mutations lead to abnormalities in soma size, dendritic arborizations, spine density, and neuronal firing that may be partially rescued by inhibiting *Pten* gene or IGF1 treatment[17-19].
ARID1B ***a,c,d***	**PI3K/AKT** **Wnt and β-catenin**	*ARID1B* suppression delays cell cycle re-entry[20,21]. Mice with *Arid1b* knock-down show suppressed IGF1, a mediator of PI3K/AKT and β-catenin pathways, resulting in decreased dendritic arborization and accumulation of aberrant dendritic spines and altered synaptic transmission[22,23].
**16p11.2** **a,d**	**RAS/ERK**	16p11.2 is one of most recurrent CNVs in ASD and encompasses multiple genes including *ERK1*. Its deletion dysregulates RAS/ERK with effect on proliferation and neurogenesis of murine neural progenitor cells[24]. 16p11.2 mutation mouse models show reduced RAS/ERK activity, reduced protein synthesis and cognitive impairments[25].
ERBIN ***a,d***	**RAS/ERK**	*ERBIN* has an inhibitory effect on RAS/ERK signaling pathway[26,27]. Up-regulation or down-regulation of *Erbin* leads to enhanced or decreased differentiation of PC12 neurons, respectively[26].
KAT2B ***a,b,c,d***	**RAS/ERK**	Null mutations lead to dysregulation of RAS/ERK and disruption of the pyramidal cell layer organization[28].
NF1 ***d***	**RAS/ERK**	Mice with *Nf1* null mutations show dysregulated RAS/ERK pathway and deficits in spine morphology, glutamate and GABA release, hippocampal LTP and learning abilities[29-32].
SYNGAP1 ***c,d***	**RAS/ERK**	*SYNGAP1* acts as RAS inhibitor, and heterozygote mutations in mice show premature neurons, elevated excitatory synaptic transmission, reduced axonal branching and synaptic boutons in inhibitory neurons, and deficits in behavior and cognition[33-35]. Rescuing *Syngap1* in adulthood did not benefit the mice, underscoring its role in prenatal and early postnatal brain development[36].
CHD8 ***a,d***	**Wnt and β-catenin**	*CHD8* knock-down disrupts G1/S phase, increases proliferation in neural progenitor and stem cells and brain overgrowth[37,38]. *Chd8* mutations in mice result in abnormalities in striatal circuitry and synaptic physiology[39].
CTNND2 ***a,c,d***	**Wnt and β-catenin**	Can affect proliferation of glioma cells and is involved in glioblastoma[40]. It regulates spine morphology[41,42]; mutations decrease spines and excitatory synapses in hippocampal neurons in rodents[43]. *Ctnnd2* mutation in mouse models have impaired spatial learning and fear conditioning[44,45].

Functional Roles in Development:
a =Neural/Glial Proliferation and Neurogenesis
b = Cell Fate and Migration
c = Neurite Outgrowth
d = Synaptogenesis and Synapse Function

References

1 Gross, C. *et al.* Increased expression of the PI3K enhancer PIKE mediates deficits in synaptic plasticity and behavior in fragile X syndrome. *Cell Rep* **11**, 727-736, doi:10.1016/j.celrep.2015.03.060 (2015).

2 Gross, C. *et al.* Selective role of the catalytic PI3K subunit p110beta in impaired higher order cognition in fragile X syndrome. *Cell Rep* **11**, 681-688, doi:10.1016/j.celrep.2015.03.065 (2015).

3 Regad, T., Roth, M., Bredenkamp, N., Illing, N. & Papalopulu, N. The neural progenitor-specifying activity of FoxG1 is antagonistically regulated by CKI and FGF. *Nat Cell Biol* **9**, 531-540, doi:10.1038/ncb1573 (2007).

4 Miyoshi, G. & Fishell, G. Dynamic FoxG1 expression coordinates the integration of multipolar pyramidal neuron precursors into the cortical plate. *Neuron* **74**, 1045-1058, doi:10.1016/j.neuron.2012.04.025 (2012).

5 Hanashima, C., Li, S. C., Shen, L., Lai, E. & Fishell, G. Foxg1 suppresses early cortical cell fate. *Science* **303**, 56-59, doi:10.1126/science.1090674 (2004).

6 Venkatesh, H. S. *et al.* Neuronal Activity Promotes Glioma Growth through Neuroligin-3 Secretion. *Cell* **161**, 803-816, doi:10.1016/j.cell.2015.04.012 (2015).

7 Chen, Y., Huang, W. C., Sejourne, J., Clipperton-Allen, A. E. & Page, D. T. Pten Mutations Alter Brain Growth Trajectory and Allocation of Cell Types through Elevated beta-Catenin Signaling. *J Neurosci* **35**, 10252-10267, doi:10.1523/JNEUROSCI.5272-14.2015 (2015).

8 Kwon, C. H. *et al.* Pten regulates neuronal arborization and social interaction in mice. *Neuron* **50**, 377-388, doi:10.1016/j.neuron.2006.03.023 (2006).

9 Huang, W. C., Chen, Y. & Page, D. T. Hyperconnectivity of prefrontal cortex to amygdala projections in a mouse model of macrocephaly/autism syndrome. *Nat Commun* **7**, 13421, doi:10.1038/ncomms13421 (2016).

10 Tamura, M. *et al.* Inhibition of cell migration, spreading, and focal adhesions by tumor suppressor PTEN. *Science* **280**, 1614-1617 (1998).

11 Marino, S. *et al.* PTEN is essential for cell migration but not for fate determination and tumourigenesis in the cerebellum. *Development* **129**, 3513-3522 (2002).

12 Baek, S. T. *et al.* An AKT3-FOXG1-reelin network underlies defective migration in human focal malformations of cortical development. *Nat Med* **21**, 1445-1454, doi:10.1038/nm.3982 (2015).

13 Bozdagi, O. *et al.* Haploinsufficiency of the autism-associated Shank3 gene leads to deficits in synaptic function, social interaction, and social communication. *Mol Autism* **1**, 15, doi:10.1186/2040-2392-1-15 (2010).

14 Yang, M. *et al.* Reduced excitatory neurotransmission and mild autism-relevant phenotypes in adolescent Shank3 null mutant mice. *J Neurosci* **32**, 6525-6541, doi:10.1523/JNEUROSCI.6107-11.2012 (2012).

15 Bozdagi, O., Tavassoli, T. & Buxbaum, J. D. Insulin-like growth factor-1 rescues synaptic and motor deficits in a mouse model of autism and developmental delay. *Mol Autism* **4**, 9, doi:10.1186/2040-2392-4-9 (2013).

16 Mellios, N. *et al.* MeCP2-regulated miRNAs control early human neurogenesis through differential effects on ERK and AKT signaling. *Mol Psychiatry*, doi:10.1038/mp.2017.86 (2017).

17 Li, Y. *et al.* Global transcriptional and translational repression in human-embryonic-stem-cell-derived Rett syndrome neurons. *Cell Stem Cell* **13**, 446-458, doi:10.1016/j.stem.2013.09.001 (2013).

18 Marchetto, M. C. *et al.* A model for neural development and treatment of Rett syndrome using human induced pluripotent stem cells. *Cell* **143**, 527-539, doi:10.1016/j.cell.2010.10.016 (2010).
19 Tropea, D. *et al.* Partial reversal of Rett Syndrome-like symptoms in MeCP2 mutant mice. *Proc Natl Acad Sci U S A* **106**, 2029-2034, doi:10.1073/pnas.0812394106 (2009).
20 Sim, J. C. *et al.* Expanding the phenotypic spectrum of ARID1B-mediated disorders and identification of altered cell-cycle dynamics due to ARID1B haploinsufficiency. *Orphanet J Rare Dis* **9**, 43, doi:10.1186/1750-1172-9-43 (2014).
21 Nagl, N. G., Jr., Wang, X., Patsialou, A., Van Scoy, M. & Moran, E. Distinct mammalian SWI/SNF chromatin remodeling complexes with opposing roles in cell-cycle control. *EMBO J* **26**, 752-763, doi:10.1038/sj.emboj.7601541 (2007).
22 Ka, M., Chopra, D. A., Dravid, S. M. & Kim, W. Y. Essential Roles for ARID1B in Dendritic Arborization and Spine Morphology of Developing Pyramidal Neurons. *J Neurosci* **36**, 2723-2742, doi:10.1523/JNEUROSCI.2321-15.2016 (2016).
23 Celen, C. *et al.* Arid1b haploinsufficient mice reveal neuropsychiatric phenotypes and reversible causes of growth impairment. *Elife* **6**, doi:10.7554/eLife.25730 (2017).
24 Pucilowska, J. *et al.* The 16p11.2 deletion mouse model of autism exhibits altered cortical progenitor proliferation and brain cytoarchitecture linked to the ERK MAPK pathway. *J Neurosci* **35**, 3190-3200, doi:10.1523/JNEUROSCI.4864-13.2015 (2015).
25 Tian, D. *et al.* Contribution of mGluR5 to pathophysiology in a mouse model of human chromosome 16p11.2 microdeletion. *Nat Neurosci* **18**, 182-184, doi:10.1038/nn.3911 (2015).
26 Huang, Y. Z., Zang, M., Xiong, W. C., Luo, Z. & Mei, L. Erbin suppresses the MAP kinase pathway. *J Biol Chem* **278**, 1108-1114, doi:10.1074/jbc.M205413200 (2003).
27 Rangwala, R., Banine, F., Borg, J. P. & Sherman, L. S. Erbin regulates mitogen-activated protein (MAP) kinase activation and MAP kinase-dependent interactions between Merlin and adherens junction protein complexes in Schwann cells. *J Biol Chem* **280**, 11790-11797, doi:10.1074/jbc.M414154200 (2005).
28 Maurice, T. *et al.* Altered memory capacities and response to stress in p300/CBP-associated factor (PCAF) histone acetylase knockout mice. *Neuropsychopharmacology* **33**, 1584-1602, doi:10.1038/sj.npp.1301551 (2008).
29 Cui, Y. *et al.* Neurofibromin regulation of ERK signaling modulates GABA release and learning. *Cell* **135**, 549-560, doi:10.1016/j.cell.2008.09.060 (2008).
30 Molosh, A. I. *et al.* Social learning and amygdala disruptions in Nf1 mice are rescued by blocking p21-activated kinase. *Nat Neurosci* **17**, 1583-1590, doi:10.1038/nn.3822 (2014).
31 Shilyansky, C. *et al.* Neurofibromin regulates corticostriatal inhibitory networks during working memory performance. *Proc Natl Acad Sci U S A* **107**, 13141-13146, doi:10.1073/pnas.1004829107 (2010).
32 Oliveira, A. F. & Yasuda, R. Neurofibromin is the major ras inactivator in dendritic spines. *J Neurosci* **34**, 776-783, doi:10.1523/JNEUROSCI.3096-13.2014 (2014).
33 Berryer, M. H. *et al.* Decrease of SYNGAP1 in GABAergic cells impairs inhibitory synapse connectivity, synaptic inhibition and cognitive function. *Nat Commun* **7**, 13340, doi:10.1038/ncomms13340 (2016).
34 Komiyama, N. H. *et al.* SynGAP regulates ERK/MAPK signaling, synaptic plasticity, and learning in the complex with postsynaptic density 95 and NMDA receptor. *J Neurosci* **22**, 9721-9732 (2002).

35 Ozkan, E. D. *et al.* Reduced cognition in Syngap1 mutants is caused by isolated damage within developing forebrain excitatory neurons. *Neuron* **82**, 1317-1333, doi:10.1016/j.neuron.2014.05.015 (2014).
36 Clement, J. P. *et al.* Pathogenic SYNGAP1 mutations impair cognitive development by disrupting maturation of dendritic spine synapses. *Cell* **151**, 709-723, doi:10.1016/j.cell.2012.08.045 (2012).
37 Sugathan, A. *et al.* CHD8 regulates neurodevelopmental pathways associated with autism spectrum disorder in neural progenitors. *Proc Natl Acad Sci U S A* **111**, E4468-4477, doi:10.1073/pnas.1405266111 (2014).
38 Cotney, J. *et al.* The autism-associated chromatin modifier CHD8 regulates other autism risk genes during human neurodevelopment. *Nat Commun* **6**, 6404, doi:10.1038/ncomms7404 (2015).
39 Platt, R. J. *et al.* Chd8 Mutation Leads to Autistic-like Behaviors and Impaired Striatal Circuits. *Cell Rep* **19**, 335-350, doi:10.1016/j.celrep.2017.03.052 (2017).
40 Schonberg, D. L., Bao, S. & Rich, J. N. Genomics informs glioblastoma biology. *Nat Genet* **45**, 1105-1107, doi:10.1038/ng.2775 (2013).
41 Arikkath, J. *et al.* Delta-catenin regulates spine and synapse morphogenesis and function in hippocampal neurons during development. *J Neurosci* **29**, 5435-5442, doi:10.1523/JNEUROSCI.0835-09.2009 (2009).
42 Brigidi, G. S. *et al.* Palmitoylation of delta-catenin by DHHC5 mediates activity-induced synapse plasticity. *Nat Neurosci* **17**, 522-532, doi:10.1038/nn.3657 (2014).
43 Turner, T. N. *et al.* Loss of delta-catenin function in severe autism. *Nature* **520**, 51-56, doi:10.1038/nature14186 (2015).
44 Israely, I. *et al.* Deletion of the neuron-specific protein delta-catenin leads to severe cognitive and synaptic dysfunction. *Curr Biol* **14**, 1657-1663, doi:10.1016/j.cub.2004.08.065 (2004).
45 Matter, C., Pribadi, M., Liu, X. & Trachtenberg, J. T. Delta-catenin is required for the maintenance of neural structure and function in mature cortex in vivo. *Neuron* **64**, 320-327, doi:10.1016/j.neuron.2009.09.026 (2009).
