# Supplementary material for: The ASD Living Biology: from cell proliferation to clinical phenotype
Source: Mol Psychiatry. 2018 Jun 22;24(1):88–107. doi: 10.1038/s41380-018-0056-y (PMC6309606; doi:10.1038/s41380-018-0056-y)
Supplement: Supplementary file 4 — Supplementary Table S4 [file 41380_2018_56_MOESM4_ESM.pdf]

Table S4. ASD-like molecular, cellular, anatomical and behavioral abnormalities in MIA models		
**Human development**	**Abnormalities induced by MIA and associated with ASD**	**Suggested Refs**
1st-2nd Trimester	Upregulated cell cycle and downregulated migration and neurite outgrowth gene expression	1, 2
	Cortical layering: over-production of neurons, increased cortical thickness, focal cortical dysplasia	3, 3,4, 5, 6
	Cerebellar vermis dysplasia	7, 8, 9, 10
2nd-3rd Trimester	Microglia: enhanced priming, activation	3,4, 11, 12, 13, 5
	Dendritic morphology abnormalities	14, 15, 16, 17
	GABAergic signaling, excitatory/inhibitory imbalance, number of interneurons	18, 19, 20, 21, 22, 23, 24
3rd Trimester - postnatal	White matter neuron density	25
	Dendritic spines number and turnover rates	26, 27
	Synaptic pruning and proteins	28, 27, 29, 30, 31, 32
Postnatal	Myelin functionality and stability	33, 34, 35
	Dopamine system	33, 36, 37, 38
	Serotonin levels	39, 40, 41
	ASD-like abnormal social, vocalization, and ritualistic behaviors	42, 3,4, 43, 44, 15, 45
	Gender-dependent effects	46
	Transgenerational effects	47, 48

References

1 Oskvig, D. B., Elkahloun, A. G., Johnson, K. R., Phillips, T. M. & Herkenham, M. Maternal immune activation by LPS selectively alters specific gene expression profiles of interneuron migration and oxidative stress in the fetus without triggering a fetal immune response. *Brain Behav Immun* **26**, 623-634, doi:10.1016/j.bbi.2012.01.015 (2012).

2 Lombardo, M. V. *et al.* Maternal immune activation dysregulation of the fetal brain transcriptome and relevance to the pathophysiology of autism spectrum disorder. *Mol Psychiatry*, doi:10.1038/mp.2017.15 (2017).

3 Smith, S. E., Elliott, R. M. & Anderson, M. P. Maternal immune activation increases neonatal mouse cortex thickness and cell density. *J Neuroimmune Pharmacol* **7**, 529-532, doi:10.1007/s11481-012-9372-1 (2012).

4 Le Belle, J. E. *et al.* Maternal inflammation contributes to brain overgrowth and autism-associated behaviors through altered redox signaling in stem and progenitor cells. *Stem Cell Reports* **3**, 725-734, doi:10.1016/j.stemcr.2014.09.004 (2014).

5 Pang, Y. *et al.* Early Postnatal Lipopolysaccharide Exposure Leads to Enhanced Neurogenesis and Impaired Communicative Functions in Rats. *PLoS One* **11**, e0164403, doi:10.1371/journal.pone.0164403 (2016).

6 Choi, G. B. *et al.* The maternal interleukin-17a pathway in mice promotes autism-like phenotypes in offspring. *Science* **351**, 933-939, doi:10.1126/science.aad0314 (2016).

7 Wu, W. L., Hsiao, E. Y., Yan, Z., Mazmanian, S. K. & Patterson, P. H. The placental interleukin-6 signaling controls fetal brain development and behavior. *Brain Behav Immun* **62**, 11-23, doi:10.1016/j.bbi.2016.11.007 (2017).

8 Aavani, T., Rana, S. A., Hawkes, R. & Pittman, Q. J. Maternal immune activation produces cerebellar hyperplasia and alterations in motor and social behaviors in male and female mice. *Cerebellum* **14**, 491-505, doi:10.1007/s12311-015-0669-5 (2015).

9 Shi, L. *et al.* Activation of the maternal immune system alters cerebellar development in the offspring. *Brain Behav Immun* **23**, 116-123, doi:10.1016/j.bbi.2008.07.012 (2009).

10 Chow, K. H., Yan, Z. & Wu, W. L. Induction of Maternal Immune Activation in Mice at Mid-gestation Stage with Viral Mimic Poly(I:C). *J Vis Exp*, e53643, doi:10.3791/53643 (2016).
11 Estes, M. L. & McAllister, A. K. Immune mediators in the brain and peripheral tissues in autism spectrum disorder. *Nat Rev Neurosci* **16**, 469-486, doi:10.1038/nrn3978 (2015).
12 Bilbo, S. D., Block, C. L., Bolton, J. L., Hanamsagar, R. & Tran, P. K. Beyond infection - Maternal immune activation by environmental factors, microglial development, and relevance for autism spectrum disorders. *Exp Neurol*, doi:10.1016/j.expneurol.2017.07.002 (2017).
13 Mattei, D. *et al.* Maternal immune activation results in complex microglial transcriptome signature in the adult offspring that is reversed by minocycline treatment. *Transl Psychiatry* **7**, e1120, doi:10.1038/tp.2017.80 (2017).
14 Weir, R. K. *et al.* Preliminary evidence of neuropathology in nonhuman primates prenatally exposed to maternal immune activation. *Brain Behav Immun* **48**, 139-146, doi:10.1016/j.bbi.2015.03.009 (2015).
15 Varghese, M. *et al.* Autism spectrum disorder: neuropathology and animal models. *Acta Neuropathol*, doi:10.1007/s00401-017-1736-4 (2017).
16 Baharnoori, M., Brake, W. G. & Srivastava, L. K. Prenatal immune challenge induces developmental changes in the morphology of pyramidal neurons of the prefrontal cortex and hippocampus in rats. *Schizophr Res* **107**, 99-109, doi:10.1016/j.schres.2008.10.003 (2009).
17 Li, W. Y., Chang, Y. C., Lee, L. J. & Lee, L. J. Prenatal infection affects the neuronal architecture and cognitive function in adult mice. *Dev Neurosci* **36**, 359-370, doi:10.1159/000362383 (2014).
18 Richetto, J. *et al.* Behavioral effects of the benzodiazepine-positive allosteric modulator SH-053-2'F-S-CH(3) in an immune-mediated neurodevelopmental disruption model. *Int J Neuropsychopharmacol* **18**, doi:10.1093/ijnp/pyu055 (2015).
19 Richetto, J., Calabrese, F., Riva, M. A. & Meyer, U. Prenatal immune activation induces maturation-dependent alterations in the prefrontal GABAergic transcriptome. *Schizophr Bull* **40**, 351-361, doi:10.1093/schbul/sbs195 (2014).
20 Klapal, L., Igelhorst, B. A. & Dietzel-Meyer, I. D. Changes in Neuronal Excitability by Activated Microglia: Differential Na(+) Current Upregulation in Pyramid-Shaped and Bipolar Neurons by TNF-alpha and IL-18. *Front Neurol* **7**, 44, doi:10.3389/fneur.2016.00044 (2016).
21 Chugh, D., Nilsson, P., Afjei, S. A., Bakochi, A. & Ekdahl, C. T. Brain inflammation induces post-synaptic changes during early synapse formation in adult-born hippocampal neurons. *Exp Neurol* **250**, 176-188, doi:10.1016/j.expneurol.2013.09.005 (2013).
22 Garcia-Oscos, F. *et al.* Activation of the anti-inflammatory reflex blocks lipopolysaccharide-induced decrease in synaptic inhibition in the temporal cortex of the rat. *J Neurosci Res* **93**, 859-865, doi:10.1002/jnr.23550 (2015).
23 Zhang, Z. & van Praag, H. Maternal immune activation differentially impacts mature and adult-born hippocampal neurons in male mice. *Brain Behav Immun* **45**, 60-70, doi:10.1016/j.bbi.2014.10.010 (2015).
24 Giovanoli, S., Weber, L. & Meyer, U. Single and combined effects of prenatal immune activation and peripubertal stress on parvalbumin and reelin expression in the hippocampal formation. *Brain Behav Immun* **40**, 48-54, doi:10.1016/j.bbi.2014.04.005 (2014).
25 Duchatel, R. J. *et al.* Increased white matter neuron density in a rat model of maternal immune activation - Implications for schizophrenia. *Prog Neuropsychopharmacol Biol Psychiatry* **65**, 118-126, doi:10.1016/j.pnpbp.2015.09.006 (2016).

26 Coiro, P. *et al.* Impaired synaptic development in a maternal immune activation mouse model of neurodevelopmental disorders. *Brain Behav Immun* **50**, 249-258, doi:10.1016/j.bbi.2015.07.022 (2015).
27 Pendyala, G. *et al.* Maternal Immune Activation Causes Behavioral Impairments and Altered Cerebellar Cytokine and Synaptic Protein Expression. *Neuropsychopharmacology* **42**, 1435-1446, doi:10.1038/npp.2017.7 (2017).
28 Forrest, C. M. *et al.* Prenatal activation of Toll-like receptors-3 by administration of the viral mimetic poly(I:C) changes synaptic proteins, N-methyl-D-aspartate receptors and neurogenesis markers in offspring. *Mol Brain* **5**, 22, doi:10.1186/1756-6606-5-22 (2012).
29 Gyorffy, B. A. *et al.* Widespread alterations in the synaptic proteome of the adolescent cerebral cortex following prenatal immune activation in rats. *Brain Behav Immun* **56**, 289-309, doi:10.1016/j.bbi.2016.04.002 (2016).
30 Fernandez de Cossio, L., Guzman, A., van der Veldt, S. & Luheshi, G. N. Prenatal infection leads to ASD-like behavior and altered synaptic pruning in the mouse offspring. *Brain Behav Immun* **63**, 88-98, doi:10.1016/j.bbi.2016.09.028 (2017).
31 Giovanoli, S., Weber-Stadlbauer, U., Schedlowski, M., Meyer, U. & Engler, H. Prenatal immune activation causes hippocampal synaptic deficits in the absence of overt microglia anomalies. *Brain Behav Immun* **55**, 25-38, doi:10.1016/j.bbi.2015.09.015 (2016).
32 Kentner, A. C., Khoury, A., Lima Queiroz, E. & MacRae, M. Environmental enrichment rescues the effects of early life inflammation on markers of synaptic transmission and plasticity. *Brain Behav Immun* **57**, 151-160, doi:10.1016/j.bbi.2016.03.013 (2016).
33 Richetto, J. *et al.* Genome-Wide Transcriptional Profiling and Structural Magnetic Resonance Imaging in the Maternal Immune Activation Model of Neurodevelopmental Disorders. *Cereb Cortex* **27**, 3397-3413, doi:10.1093/cercor/bhw320 (2017).
34 Farrelly, L. *et al.* Maternal immune activation induces changes in myelin and metabolic proteins, some of which can be prevented with risperidone in adolescence. *Dev Neurosci* **37**, 43-55, doi:10.1159/000368305 (2015).
35 Makinodan, M. *et al.* Maternal immune activation in mice delays myelination and axonal development in the hippocampus of the offspring. *J Neurosci Res* **86**, 2190-2200, doi:10.1002/jnr.21673 (2008).
36 Luchicchi, A. *et al.* Maternal Immune Activation Disrupts Dopamine System in the Offspring. *Int J Neuropsychopharmacol* **19**, doi:10.1093/ijnp/pyw007 (2016).
37 Baharnoori, M., Bhardwaj, S. K. & Srivastava, L. K. Effect of maternal lipopolysaccharide administration on the development of dopaminergic receptors and transporter in the rat offspring. *PLoS One* **8**, e54439, doi:10.1371/journal.pone.0054439 (2013).
38 Ito, H. T., Smith, S. E., Hsiao, E. & Patterson, P. H. Maternal immune activation alters nonspatial information processing in the hippocampus of the adult offspring. *Brain Behav Immun* **24**, 930-941, doi:10.1016/j.bbi.2010.03.004 (2010).
39 Hsueh, P. T. *et al.* Expression of cerebral serotonin related to anxiety-like behaviors in C57BL/6 offspring induced by repeated subcutaneous prenatal exposure to low-dose lipopolysaccharide. *PLoS One* **12**, e0179970, doi:10.1371/journal.pone.0179970 (2017).
40 Holloway, T. *et al.* Prenatal stress induces schizophrenia-like alterations of serotonin 2A and metabotropic glutamate 2 receptors in the adult offspring: role of maternal immune system. *J Neurosci* **33**, 1088-1098, doi:10.1523/JNEUROSCI.2331-12.2013 (2013).
41 Wischhof, L., Irrsack, E., Dietz, F. & Koch, M. Maternal lipopolysaccharide treatment differentially affects 5-HT(2A) and mGlu2/3 receptor function in the adult male and female rat offspring. *Neuropharmacology* **97**, 275-288, doi:10.1016/j.neuropharm.2015.05.029 (2015).

42 Malkova, N. V., Yu, C. Z., Hsiao, E. Y., Moore, M. J. & Patterson, P. H. Maternal immune activation yields offspring displaying mouse versions of the three core symptoms of autism. *Brain Behav Immun* **26**, 607-616, doi:10.1016/j.bbi.2012.01.011 (2012).

43 Bauman, M. D. *et al.* Activation of the maternal immune system during pregnancy alters behavioral development of rhesus monkey offspring. *Biol Psychiatry* **75**, 332-341, doi:10.1016/j.biopsych.2013.06.025 (2014).

44 Machado, C. J., Whitaker, A. M., Smith, S. E., Patterson, P. H. & Bauman, M. D. Maternal immune activation in nonhuman primates alters social attention in juvenile offspring. *Biol Psychiatry* **77**, 823-832, doi:10.1016/j.biopsych.2014.07.035 (2015).

45 Labouesse, M. A., Langhans, W. & Meyer, U. Long-term pathological consequences of prenatal infection: beyond brain disorders. *Am J Physiol Regul Integr Comp Physiol* **309**, R1-R12, doi:10.1152/ajpregu.00087.2015 (2015).

46 Xuan, I. C. & Hampson, D. R. Gender-dependent effects of maternal immune activation on the behavior of mouse offspring. *PLoS One* **9**, e104433, doi:10.1371/journal.pone.0104433 (2014).

47 Weber-Stadlbauer, U. *et al.* Transgenerational transmission and modification of pathological traits induced by prenatal immune activation. *Mol Psychiatry* **22**, 102-112, doi:10.1038/mp.2016.41 (2017).

48 Ronovsky, M. *et al.* Maternal immune activation transgenerationally modulates maternal care and offspring depression-like behavior. *Brain Behav Immun* **63**, 127-136, doi:10.1016/j.bbi.2016.10.016 (2017).
